# Wuhu decoction combined with azithromycin for treatment of *Mycoplasma* pneumoniae pneumonia in Asian children: a systematic review and meta analysis of randomized controlled trials

**DOI:** 10.3389/fphar.2024.1329516

**Published:** 2024-04-03

**Authors:** Shuo Yang, Xinying Liu, Huizhe Wang, Haokai Wang, Dan Sun, Yaowei Han, Huanmin Li, Xinmin Li

**Affiliations:** ^1^ Department of Pediatrics, First Teaching Hospital of Tianjin University of Traditional Chinese Medicine, Tianjin, China; ^2^ National Clinical Research Center for Chinese Medicine Acupuncture and Moxibustion, Tianjin, China

**Keywords:** Wuhu decoction, *Mycoplasma* pneumoniae pneumonia, systematic review, meta analysis, clinical research

## Abstract

**Objective:** This study constitutes a pioneering systematic review and meta analysis delving into the clinical efficacy and safety of the combined therapy involving Wuhu Decoction and azithromycin for treating *Mycoplasma* pneumoniae pneumonia in pediatric patients.

**Methods:** This study conducted a comprehensive computerized search, covering 6 Chinese databases and 6 English databases, to collect randomized controlled trials related to the combined use of Wuhu Decoction and azithromycin for treating *Mycoplasma* pneumoniae pneumonia in pediatric patients. The search was extended until August 2023. Two independent researchers were involved in literature screening, data extraction, and bias risk assessment. Meta-analysis was performed using Stata 14.0 and RevMan 5.4 software. Additionally, meta-regression analysis and subgroup analysis were carried out on primary outcomes to identify potential sources of heterogeneity and confounding factors.

**Results:** A total of 22 randomized controlled trials involving 2,026 patients were included in this study. The combined therapy of Wuhu Decoction and azithromycin demonstrated superior efficacy compared to azithromycin alone (RR = 1.17, 95% CI [1.13, 1.21], *p* < 0.00001; low certainty of evidence). Additionally, patients receiving the combination therapy experienced significantly reduced the disappearance time of fever (MD = −1.42, 95% CI [−1.84, −1.00], *p* < 0.00001; very low certainty of evidence), disappearance time of cough (MD = −2.08, 95% CI [−2.44, −1.71], *p* < 0.00001; very low certainty of evidence), disappearance of pulmonary rales (MD = −1.97, 95% CI [−2.31, −1.63], *p* < 0.00001; very low certainty of evidence), and disappearance time of wheezing (MD = −1.47, 95% CI [−1.72, −1.22], *p* < 0.00001; very low certainty of evidence). Meta-regression analysis suggested that course of disease, sample size, and age might be sources of heterogeneity. Subgroup and sensitivity analyses reaffirmed the stability of these results. Furthermore, analyses of secondary outcomes such as T lymphocytes, serum inflammatory factors, and the incidence rate of adverse reactions consistently favored the combination therapy of WHD and azithromycin over azithromycin alone, with statistically significant differences.

**Conclusion:** Based on our meta-analysis findings, the combined therapy of Wuhu Decoction and azithromycin for treating pediatric *Mycoplasma* pneumoniae pneumonia exhibited superior overall efficacy in comparison to azithromycin monotherapy. However, in the included 22 studies, the majority of evaluated factors showed unclear bias risks, and a persistent bias risk was consistently present within one category. Moreover, due to the low quality of evidence, interpreting these results should be approached with caution. Hence, we emphasize the necessity for future high-quality, multicenter, and large-sample clinical randomized controlled trials. These trials are essential to provide more robust data for evidence-based research and to establish higher-quality evidence support.

**Systematic Review Registration:** https://www.crd.york.ac.uk/prospero/, identifier CRD42023465606

## 1 Introduction

*Mycoplasma* pneumoniae (MP) is a cell wall-free prokaryote and a significant causative agent of both upper and lower respiratory tract infections (Bradley et al., 2011; Hao et al., 2022). *Mycoplasma* pneumoniae pneumonia (MPP) represents a respiratory and pulmonary inflammatory disease resulting from MP infection. It predominantly affects children between the ages of 3 and 15, primarily those in preschool and school-aged groups. This condition is characterized by its high incidence, prolonged duration, and susceptibility to recurring episodes (Gao et al., 2019; Kutty et al., 2019). Due to the rapid growth and development during this phase, children’s immune systems are not yet fully mature, rendering them more vulnerable to MP infections (Saraya et al., 2014). Epidemiological studies indicate that MPP occurs periodically, with outbreaks happening every 3–7 years and lasting for several months to a year. In certain regions, MP is the most common pathogen responsible for community-acquired pneumonia (CAP) in children, contributing to more than 40% of cases. Approximately 18% of affected children require hospitalization, underscoring its significance as a major global health concern in pediatric healthcare (Nir-Paz et al., 2017; Hançerli Demirbaş et al., 2023). The pathogenesis of MPP is intricate, involving direct damage caused by MP adhesion and toxicity, as well as disruptions in the host’s immune function. Clinical symptoms of MPP are typically atypical. In the initial stages, children commonly exhibit an irritating dry cough and fever, accompanied by symptoms like wheezing, fatigue, chills, headache, and substernal pain. As the condition progresses, complications such as pleural effusion, pulmonary embolism, atelectasis, myocarditis, hemolytic anemia, and meningitis may arise (Nir-Paz et al., 2017). Due to the complexity of clinical diagnosis for MPP, the disease is frequently misdiagnosed as viral or bacterial respiratory infections (Waites et al., 2017).

In contemporary clinical practice, macrolide antibiotics are extensively employed for the treatment of MPP in children. Among these, azithromycin stands out due to its prolonged half-life, high blood concentration, and excellent tissue penetration, making it a commonly preferred choice (Hao et al., 2022). However, owing to the underdeveloped immune system and respiratory system in children, inflammatory responses might persist, necessitating a relatively prolonged duration of macrolide antibiotic therapy. Research literature indicates that adverse reactions to azithromycin in children under 10 years old can have an incidence as high as 24.4%. Common digestive tract reactions observed in patients encompass abdominal pain, nausea, vomiting, diarrhea, potential impairment of liver and kidney function, along with neurological complications (Li and Sun, 2018; Liao et al., 2021). The widespread usage of macrolide drugs has led to the gradual development of resistance in MP, creating a new challenge in the field of antibiotic treatment. In recent years, there has been a global increase in reports of *Mycoplasma* pneumoniae resistance, particularly in the Asia-Pacific region (Cui and Liao, 2020). Strains resistant to macrolide antibiotics have been isolated from patients with MPP in Japan. In certain regions, the resistance rate of MP has surpassed 90% (Uh et al., 2013; Guo et al., 2019). This alarming rise in antibiotic resistance has significantly complicated drug treatments, making them more difficult and intricate. Furthermore, the proportion of cases that are challenging to treat and severe is continually escalating.

The prevalence of macrolide resistance in Asia stands at approximately 80%, a significantly higher rate compared to the relatively lower resistance observed in Europe and the United States (Cao et al., 2010; Ferguson et al., 2013; Zheng et al., 2015). Traditional Chinese Medicine boasts centuries of practical experience in effectively treating pneumonia (Lin et al., 2019). Capitalizing on these advantages, traditional Chinese medicine has become a focal point of research in China. Studies have affirmed that TCM can modulate inflammatory factors in a non-specific, multi-target manner, enhance the immune system, and decrease cell apoptosis (Yang et al., 2017).

In this context, the integration of traditional Chinese medicine with conventional treatments has been implemented in clinical practice to reduce reliance on antibiotics and enhance efficacy in managing MPP. Wuhu Decoction (WHD), a renowned formula for treating pneumonia, finds its origins in the Song Dynasty’s “Renzhai Zhizhi Fanglun.” This formulation comprises seven botanical drugs (Table 1) and possesses functions such as clearing heat, resolving phlegm, soothing the lungs, and relieving asthma. The method of preparation involves boiling seven botanical drugs together for 30 min, and the resulting decoction is known as Wuhu Decoction. The nomenclature of medicinal plants has been verified and corrected based on the Medicinal Plant Names Services (e.g., http://mpns.kew.org/mpns-portal/). In addition, we referred to the Chinese Pharmacopoeia 2020 regarding the names of non-botanical drugs. The relevant information about WHD referred to the original study and the Chinese Pharmacopoeia 2020. The details are shown in Supplementary Table S1. Modern pharmacological studies have demonstrated that WHD exerts anti-inflammatory, bronchodilatory, immunomodulatory, antiviral, and other effects. As the medicine recommended by the “Expert Consensus on Diagnosis and Treatment of Integrated Traditional Chinese and Western Medicine of MP in Children (formulated in 2017)” (Liu and Ma, 2017), WHD has been extensively employed in the treatment of *Mycoplasma* pneumonia in Chinese children, showing improvements in clinical efficacy, inhibition of inflammatory mediators in serum, enhancement of lung function, improvement of immune function, and reduction of adverse reactions. Leveraging a substantial number of randomized controlled trials (RCTs), this study aims to systematically assess the effectiveness and safety of WHD in pediatric MPP treatment. Finally evidence-level assessments will be conducted, providing a scientific foundation and reference for the clinical management of pediatric MPP (Chen et al., 2023).

**TABLE 1:** Ingredients and basic information of WHD.

Botanical drug	Latin name	Family
*Ephedra sinica* Stapf	Ephedra Herba	Ephedraceae
*Prunus armeniaca* L.	Amygdalus Communis Vas	Rosaceae
*Glycyrrhiza glabra* L.	Licorice	Fabaceae
*Ilex pubescens* Hook. & Arn.	Camellia sinensis O. Ktze.	Aquifoliaceae
*Pinellia ternata* (Thunb.) Makino	Arum Ternatum Thunb.	Araceae
*Morus alba* L.	Mori Cortex	Moraceae
Gypsum	Gypsum Fibrosum	Mineral drugs

## 2 Materials and methods

### 2.1 Protocol and registration

This study adhered to the guidelines outlined in the Preferred Reporting Items for Systematic Reviews and Meta-Analyses (PRISMA) guidelines (Hutton et al., 2015; Shamseer et al., 2015), as shown in Supplementary Table S1. Our study protocol was registered and published on PROSPERO, with the registration number CRD42023465606. Ethical approval was not required to conduct this study, as all data were derived from published studies.

### 2.2 Literature source and search strategy

The search methodology involved a comprehensive exploration of both English and Chinese databases. The English databases comprised Cochrane Library, PubMed, Embase, Web of Science, Ovid, and Medline database. The Chinese databases encompassed CNKI, Wanfang Data, VIP, CBM, Duxiu Database, and Yiigle. The search extended until August 2023, encompassing all accessible literature in each database. Our search strategy amalgamated specific subject terms and free-text terms tailored to the unique attributes of each database. Additionally, we conducted supplementary manual searches of the references included in the studies and reached out to pertinent authors to procure unpublished data. Furthermore, we systematically explored clinical trial registries to identify publicly available studies pertinent to the combination therapy of WHD and azithromycin in the pediatric MPP treatment. Taking the English database search as an example, the detailed search strategy was shown in Supplementary Table S2.

### 2.3 Eligibility criteria

#### 2.3.1 Study type

Randomized controlled trials (RCTs).

#### 2.3.2 Research topic

The study focuses on the combined use of WHD and azithromycin in treating pediatric MPP.

#### 2.3.3 Participants

Children diagnosed with MPP through laboratory tests and imaging examinations.

#### 2.3.4 Interventions

The experimental group received the intervention of WHD combined with the interventions utilized in the control group. The control group received azithromycin alone (or combined with CT/SHLG/AA).

#### 2.3.5 Statistical methods

Studies were required to employ appropriate statistical methods. There were no restrictions on whether blinding and allocation concealment were implemented.

#### 2.3.6 Observation index

Primary Outcome Measures: 1) Total Effective Rate: The effectiveness was determined based on improvements or worsening of a child’s clinical symptoms and objective indicators. The Total Effective Rate was calculated using the formula: (Total number of patients - Number of ineffective patients)/Total number of patients. 2) Time to Disappearance of Clinical Symptoms: This includes fever, cough, lung rales, and wheezing.

Secondary Outcome Measures: 1) Length of Hospital Stay: Duration of the hospital stay for each patient. 2) Serum Inflammatory Factors: Levels of C-reactive protein (CRP), Interleukin-4 (IL-4), Interleukin-6 (IL-6), Interleukin-8 (IL-8), Interleukin-10 (IL-10), and Tumor Necrosis Factor-alpha (TNF-α) in the bloodstream. 3) T Lymphocyte Subsets: Analysis of CD3^+^ T lymphocytes (CD3^+^), CD4^+^ T lymphocytes (CD4^+^), CD8^+^ T lymphocytes (CD8^+^), and the CD4+/CD8+ T lymphocyte ratio (CD4+/CD8+). 4) Safety: This encompasses the incidence rates of adverse events (AEs) and adverse reactions (ADRs).

### 2.4 Excluded criteria

1) Non-randomized Controlled Trials: Only RCTs were considered, excluding other types of clinical studies to ensure methodological consistency. 2) Duplicate Publications: Studies previously published elsewhere were excluded to prevent duplication and maintain data integrity. 3) Reviews and Animal Experiments: Literature types such as reviews, cell experiments, animal experiments, and articles unrelated to the research topic were excluded for relevance and specificity. 4) Data Accuracy: Studies with incomplete or erroneous data, or those with statistical method errors, were excluded to uphold data accuracy and reliability. 5) Unavailable Full Text: Studies lacking complete research documents were excluded due to unavailability of comprehensive information. 6) Exclusion of Other Antibiotics: Studies utilizing antibiotics other than WHD in combination with azithromycin for intervention were excluded to maintain uniformity in the intervention approach. 7) Exclusion of Severe Cases: Clinical cases diagnosed as severe *Mycoplasma* pneumoniae pneumonia or refractory *Mycoplasma* pneumoniae pneumonia were excluded from the analysis.

### 2.5 Literature screening and data extraction

Following the predefined inclusion and exclusion criteria, two researchers (SY and YH) independently conducted a comprehensive literature search across 12 databases. They performed initial screening based on titles and abstracts, excluding studies that were evidently unrelated to the research topic. Subsequently, the selected literature underwent a full-text review to determine inclusion or exclusion from the study. For data extraction, the researchers utilized Excel software and recorded the following information: 1) Basic Characteristics of Included Studies: Title, First Author, Publication Year, Area, Study Duration, Sample Size; 2) Study Subjects’ Information: Gender, Age, Length of Hospital Stay, Course of disease; 3) Intervention Measures: WHD combined with azithromycin/Azithromycin alone; 4) Outcome Indicators. Any discrepancies in the screening and extraction results were resolved through discussion, verification, or consultation with a third researcher (XL) to ensure accuracy and consistency in the data collection process.

### 2.6 Risk of bias assessment

Two researchers (HW and DS) independently employed the risk of bias assessment tool from Cochrane handbook (Higgins et al., 2011) to evaluate the risk of bias in the included studies. Their assessments were meticulously cross-verified to ensure accuracy. The assessment criteria encompassed multiple aspects, including random sequence generation, allocation concealment, blinding of both researchers and participants, blinding of outcome assessors, completeness of outcome data, selective reporting, and other potential biases. Each included study underwent a detailed evaluation, with assessments made item by item and categorized into three levels: low risk, unclear risk, and high risk, in accordance with the predefined criteria for risk of bias assessment. Any disparities in the assessments conducted by the two researchers were resolved through consultation with a third researcher (XL) to ensure consensus.

### 2.7 Statistical analysis

Comprehensive statistical analyses were performed on the extracted data employing RevMan 5.4 software and Stata 16.0 software. Dichotomous data, such as total effective rate and adverse event incidence rate, were primarily assessed using risk ratio (RR), accompanied by corresponding 95% confidence interval (95% CI). Meanwhile, mean differences (MD) and their 95% CI were utilized for the analysis results of other continuous outcome measures. To investigate potential sources of heterogeneity, meta-regression analyses were conducted, considering six key factors: age, course of disease, geographical region (North or South China), sample size, drug dosage, and acupoint application. Additionally, sensitivity analyses were executed to assess heterogeneity and enhance result stability. The Grading of Recommendations, Assessment, Development, and Evaluation (GRADE) approach was employed to evaluate the certainty of evidence for primary outcomes and safety. Data analysis was performed using RevMan 5.4 and R language, while risk of bias assessment utilized Cochrane tools.

## 3 Results

### 3.1 Study selection

**FIGURE 1:**
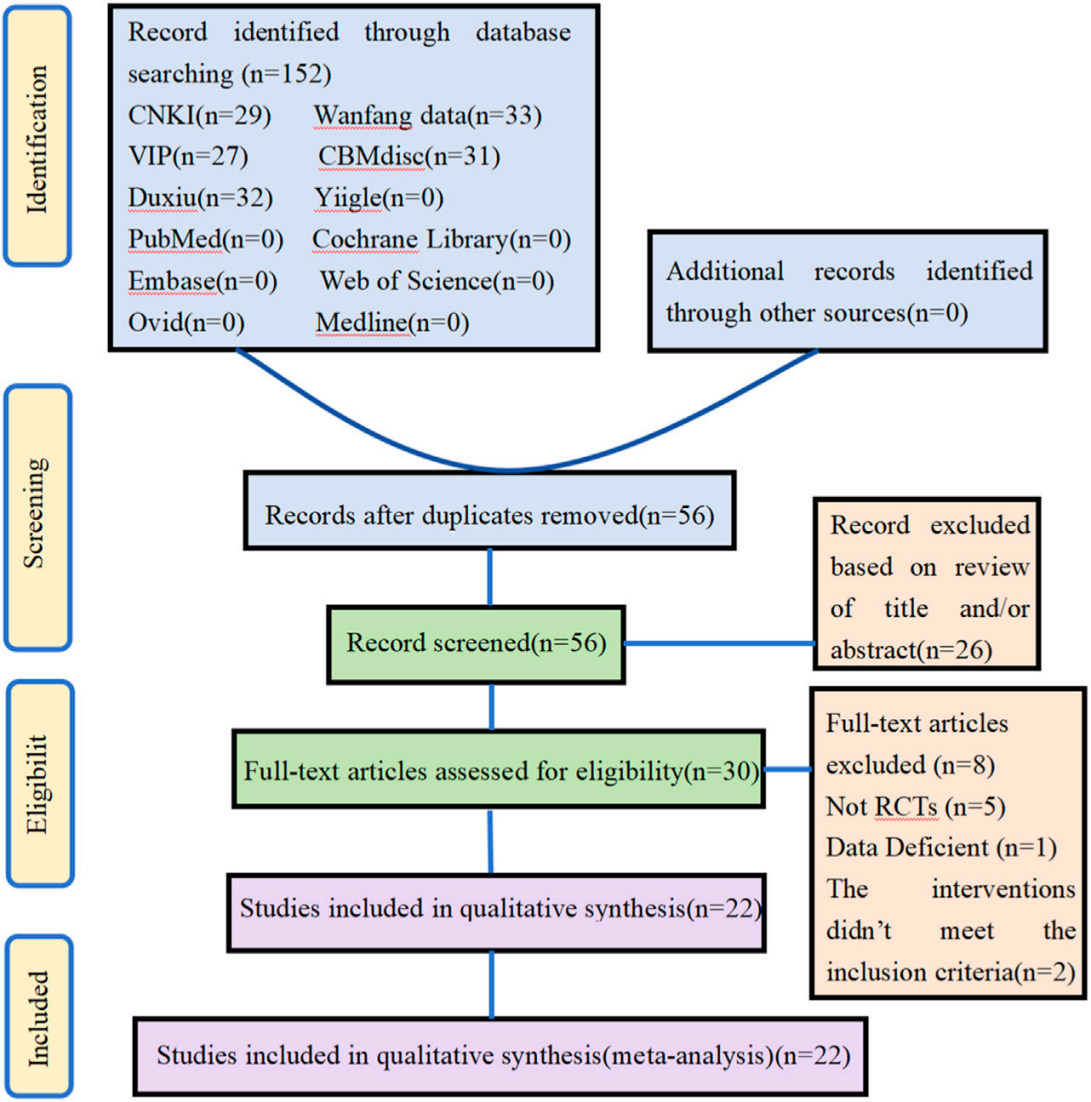
Flow chart of literature screening.

In the initial screening, searches were performed in 6 Chinese databases and 6 English databases, yielding 152 articles. Additional searches for references from relevant studies did not yield any eligible literature. After removing 96 duplicate studies in the initial screening, 56 candidate articles remained. Subsequently, the titles and abstracts of these articles were screened, resulting in 30 studies. Following this, full-text screening of these 30 studies was conducted based on the same inclusion criteria, leading to the exclusion of eight studies. Through a detailed hierarchical screening process, 22 studies were ultimately included. The flowchart outlining the literature selection process and providing detailed information on the screening results is presented in Figure 1.

### 3.2 Studies and characteristics

This study encompassed 22 selected studies (Wang, 2015; Xi et al., 2015; Liu, 2016; Su, 2016; Liu and Yao, 2017; Bian et al., 2018; Dong et al., 2018; Wang et al., 2018; Wu and Jia, 2018; Chen, 2019; Chen and Liang, 2019; Qi, 2019; Yan, 2019; Li and Wang, 2020; Ren and Yuan, 2020; Deng et al., 2021; Qi, 2021; Li et al., 2022; Ma and Feng, 2022; Shi and Cui, 2022; Gong, 2023; Yang, 2023), involving a total of 2026 children. The age range of these participants spanned from 1 to 14 years, with course of disease varying from 1 to 16 days. The treatment duration across the studies ranged from 7 to 21 days. In the experimental group, the primary treatment interventions comprised the combination of WHD with azithromycin. Specifically, this included WHD combined with azithromycin in 21 studies and WHD granules (WHDG) combined with azithromycin in 1 study. In the control group, all 22 studies utilized azithromycin as the sole intervention. Of these, 15 studies employed intravenous administration of azithromycin, while the remaining seven studies opted for oral administration. The dosages of WHD varied from 100 mL to 1000 mL daily. Azithromycin dosages were diverse, encompassing sequential therapy involving intravenous injection at 10 mg/(kg.d) and subsequent oral treatment at 10 mg/(kg.d), as well as independent oral treatment at 10 mg/(kg.d) (Supplementary Table S3).

### 3.3 Methodological quality

**FIGURE 2:**
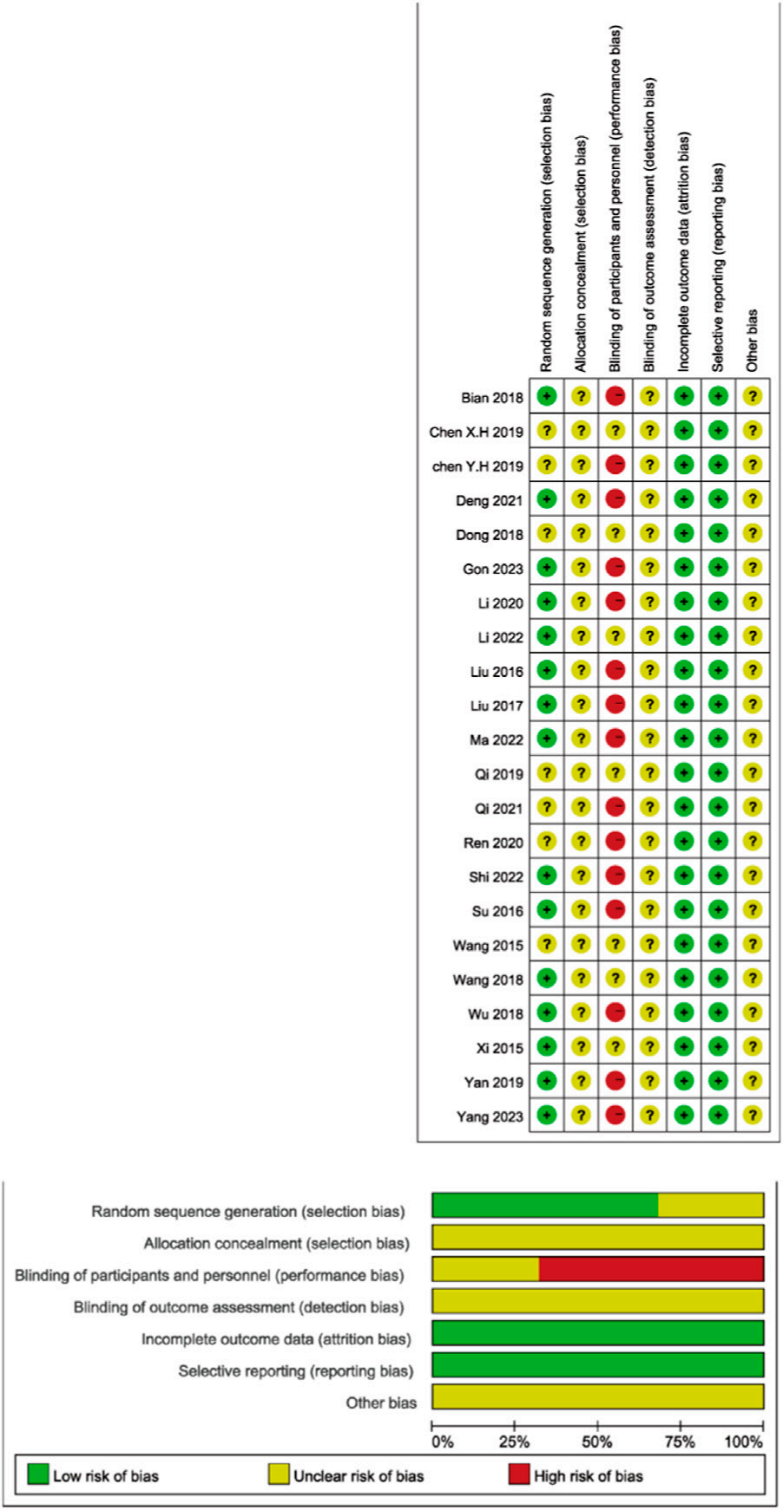
Literature quality evaluation plots.

In the pool of 22 studies, all were RCTs. Out of these, 15 studies (68.18%) meticulously outlined their procedures for generating random sequences, primarily employing random number tables. The rest of the studies asserted the use of random methods but did not specify the particular techniques used. None of the studies made reference to allocation concealment or other possible biases. Within these trials, 15 studies (68.18%) explicitly stated that blinding was not implemented for both researchers and participants. However, the remaining studies did not clearly articulate whether blinding was applied to researchers, participants, and outcome assessors (Figure 2).

### 3.4 Results of the meta-analysis

#### 3.4.1 Primary outcomes

##### 3.4.1.1 Response rate

**FIGURE 3:**
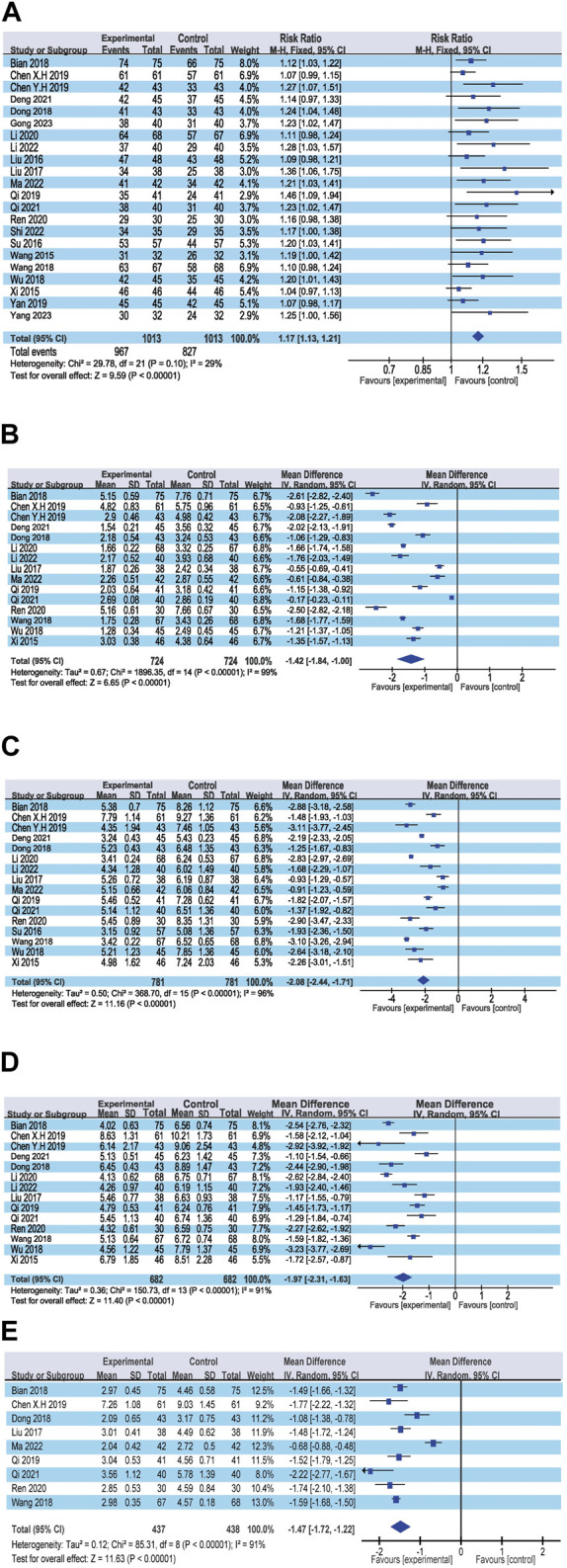
Forest plot of the primary outcomes. **(A)** Response rate; **(B)** Disappearance time of fever; **(C)** Disappearance time of cough; **(D)** Disappearance time of pulmonary rale; **(E)** Disappearance time of wheezing.

**FIGURE 4:**
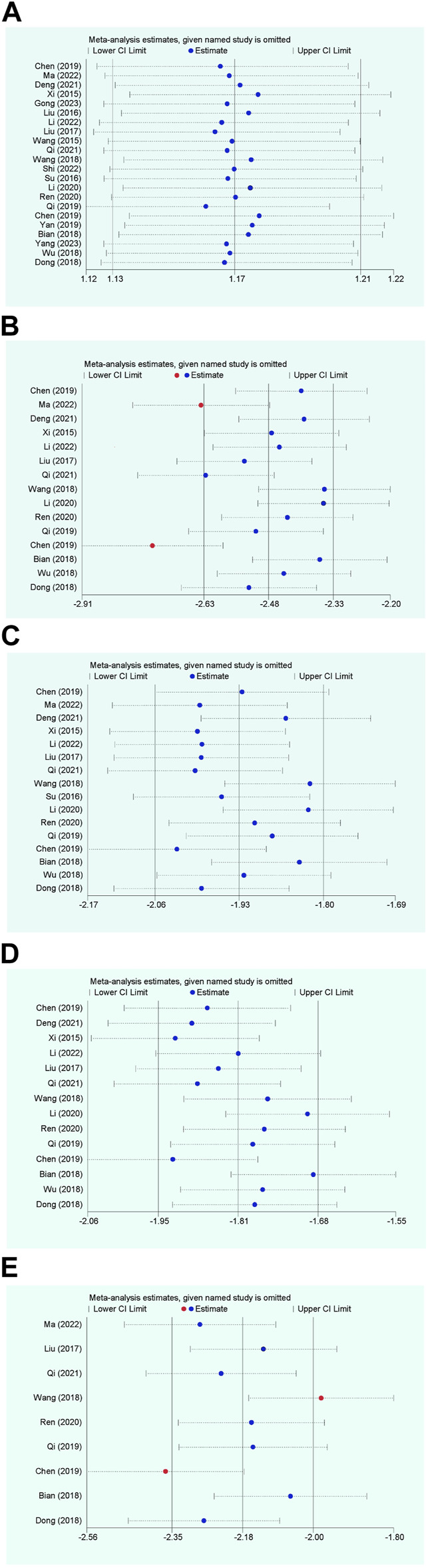
Sensitivity analysis plot. **(A)** Response rate; **(B)** Disappearance time of fever; **(C)** Disappearance time of cough; **(D)** Disappearance time of pulmonary rale; **(E)** Disappearance time of wheezing.

The overall efficacy rate was documented across 22 RCTs, encompassing 2026 patients. In the cohort treated with modified WHD combined with azithromycin, out of 1,013 patients, 967 exhibited effectiveness, yielding an efficacy rate of 95.45%. In the control group, where patients were administered azithromycin alone, out of 1,013 patients, 827 showed effectiveness, resulting in an overall efficacy rate of 81.64%. Meta-analysis findings showcased a significant disparity in the overall efficacy rates between the two groups (RR = 1.17, 95% CI [1.13, 1.21], *p* < 0.00001). The heterogeneity test revealed an I^2^ value of 29%, indicating relatively minor heterogeneity among the studies. Consequently, a fixed-effects model was employed. However, due to the presence of some heterogeneity, the certainty of the evidence was deemed low (Figure 3A; Table 2). Multivariate meta-regression analysis outcomes suggested that factors such as age, dosage, sample size, regional disparities between North and South China, course of disease, and the use of acupoint application did not significantly interact and could not entirely account for the observed heterogeneity (Supplementary Figure S1; Table 3). Differences in assessment methods and standards across studies were identified as potential sources of the observed heterogeneity, as indicated by sensitivity analysis, which demonstrated relatively stable results (Figure 4A).

**TABLE 2:** Grading of recommendations, assessment, development, and evaluation of primary outcomes and safety of WHD+Azithromycin compared with Azithromycin.

Outcomes	Detail of outcomes	Studies	Sample size (T/C)	Relative effect (95%CI)	Quality of evidence^a^ (GRADE assessment)
Primary Outcomes	Response rate	22	1,013/1,013	RR 1.17 (1.13, 1.21)	Low^1,6^
	Disappearance time of fever	15	724/724	MD −1.42 (−1.84, −1.00)	Very Low^1,3^
	Disappearance time of pulmonary rale	14	682/682	MD −1.97 (−2.31, −1.63)	Very Low^1,3^
	Disappearance time of wheezing	9	437/438	MD −1.47 (−1.72, −1.22)	Very Low^1,3^
	Disappearance time of cough	16	781/781	MD −2.08 (−2.44, −1.71)	Very Low^1,3^
Safety	Adverse rate	8	430/430	RR 0.42 (0.27, 0.67)	Low^1,6^

RR, odds ratio; MD, mean difference; 95% CI, 95% Credible Interval; GRADE, grading of recommendations, Assessment, Development, and Evaluation.

Interventions: WHD, Wuhu decoctionx.

^a^ Estimates for primary outcomes and safety with the Grading of Recommendations, Assessment, Development, and Evaluation Assessment: 1. downgraded because of risk of bias; 2. downgraded one levels because of inconsistency; 3. downgraded two levels because of inconsistency; 4. downgraded because of indirectness; 5. downgraded because of imprecision; 6. downgraded because of publication bias; 7. Upgraded because of Large effects, confounding factors, and quantity-effect relationship.

**TABLE 3:** The meta-regression results of primary outcomes.

	Category of regression^a^	Estimate	Pval	95% CI
Response rate	Sample size	−0.0009358	0.145	[−0.0022216, 0.0003501]
	Age	0.0115788	0.431	[−0.018612, 0.0417695]
	Dose	−0.0000903	0.235	[−0.0002455, 0.0000649]
	Southern regions or northern regions	−0.0205886	0.555	[−0.0920988, 0.0509217]
	Course of disease	0.014221	0.217	[−0.0093921, 0.037834]
	Whether to add CMS (such as acupoint application)	−0.0209887	0.602	[−0.1036112, 0.0616337]
Disappearance time of fever	Sample size	−0.0185587	0.007	[−0.0311477, −0.0059696]**
	Age	0.0069173	0.979	[−0.5529241, 0.5667588]
	Dose	−0.0020539	0.695	[−0.0133924, 0.0092845]
	Southern regions or northern regions	−0.1363747	0.730	[−0.9728331, 0.7000837]
	Course of disease	−0.2134666	0.025	[−0.3933086, −0.0336245]*
	Whether to add CMS (such as acupoint application)	0.2702478	0.492	[−0.5547017, 1.0951197]
Disappearance time of cough	Sample size	−0.0104757	0.145	[−0.0250219, 0.0040705]
	Age	0.1364232	0.546	[−0.3392176, 0.612064]
	Dose	−0.0038138	0.479	[−0.0152763, 0.0076488]
	Southern regions or northern regions	0.731455	0.862	[−0.8136033, 0.9598943]
	Course of disease	0.0344628	0.759	[−0.209231, 0.2781565]
	Whether to add CMS (such as acupoint application)	0.3069358	0.464	[−0.5674663, 1.181338]
Disappearance time of pulmonary rale	Sample size	−0.0047978	0.498	[−0.0197435, 0.0101478]
	Age	0.3843905	0.034	[0.0338157, 0.7349654]*
	Dose	−0.0031888	0.475	[−0.0128719, 0.0064943]
	Southern regions or northern regions	0.2291565	0.551	[−0.5841731, 1.042486]
	Course of disease	−0.019858	0.854	[−0.2578703, 0.2181543]
	Whether to add CMS (such as acupoint application)	−0.2604899	0.521	[−1.118195, 0.597215]
Disappearance time of wheezing	Sample size	−0.2216008	0.495	[−0.9498865, 0.5066849]
	Age	−0.2216008	0.495	[−0.9498865, 0.5066849]
	Dose	0.0005613	0.865	[−0.0075049, 0.0086275]
	Southern regions or northern regions	−0.0009771	0.854	[−0.0130782, 0.011124]
	Course of disease	0.1671403	0.037	[0.0145114, 0.3197693]*
	Whether to add CMS (such as acupoint application)	0.5213482	0.112	[−0.1562355, 1.198932]

^a^ Category of regression were the covariate that may affect our results in this manuscript.

Pval: *p*-value; 95% CI, 95% confidence interval.

Significant codes

(**): Pval (0.001–0.01).

(*): Pval (0.01–0.05).

##### 3.4.1.2 Disappearance time of fever

Fifteen RCTs contributed data on fever resolution time, encompassing a total of 1,448 patients. A statistically significant distinction in fever resolution time emerged between the group treated with WHD combined with azithromycin and the group receiving azithromycin alone. However, the certainty of this evidence was very low (MD = −1.42, 95% CI [−1.84, −1.00], *p* < 0.00001) (Figure 3B; Table 2). Notably, considerable heterogeneity was observed among the studies (*p* < 0.00001, I^2^ = 99%), prompting a meta-regression analysis to explore potential sources of this heterogeneity. The multivariate meta-regression analysis suggested that estimated values increased with the rise in sample size and course of disease (Supplementary Figure S2). Specifically, sample size (estimate = −0.0185587, *p* = 0.007, 95% CI [−0.0311477, −0.0059696]) and course of disease (estimate = −0.2134666, *p* = 0.025, 95% CI [−0.3933086, −0.0336245]) were identified as potential sources of heterogeneity (Table 3). Subgroup analysis further corroborated that sample size (*p* = 0.0009, I^2^ = 85.7%) and course of disease (*p* = 0.01, I^2^ = 83.2%) were sources of heterogeneity, although they did not compromise result stability (Supplementary Table S4 and Supplementary Figures S3A, B). Sensitivity analysis pinpointed two studies (Chen and Liang, 2019; Ma and Feng, 2022) as potential sources of heterogeneity. Excluding these studies altered the results (MD = −1.43, 95% CI [−1.89, −0.98]) but did not jeopardize result stability (Figure 4B).

##### 3.4.1.3 Disappearance time of cough

Sixteen RCTs contributed data on cough resolution time, encompassing a total of 1,562 patients. A statistically significant disparity in cough resolution time emerged between the group treated with WHD combined with azithromycin and the group receiving azithromycin alone, although the certainty of this evidence was very low (MD = −2.08, 95% CI [−2.44, −1.71], *p* < 0.00001) (Figure 3C; Table 2). Notably, considerable heterogeneity was observed among the studies (*p* < 0.00001, I^2^ = 96%). Consequently, a random-effects model was employed for the analysis, and efforts were made to explore potential sources of heterogeneity through meta-regression analysis. The multivariate meta-regression analysis suggested that interactions among age, dosage, sample size, regional disparities between North and South China, course of disease, and the use of acupoint application were absent. Consequently, these factors could not comprehensively elucidate the observed heterogeneity (Supplementary Figure S4; Table 3). Sensitivity analysis indicated the relative stability of our results; even after excluding one publication, our conclusions remained unaltered (Figure 4C).

##### 3.4.1.4 Disappearance time of pulmonary rale

Fourteen RCTs supplied data on the disappearance time of pulmonary rales, encompassing a total of 1,364 patients. We observed a statistically significant distinction in the disappearance time of pulmonary rales between the group treated with WHD combined with azithromycin and the group receiving azithromycin alone. However, the certainty of this evidence was relatively low (MD = −1.97, 95% CI [−2.31, −1.63], *p* < 0.00001) (Figure 3D; Table 2). Considerable heterogeneity was detected among the studies (*p* < 0.00001, I^2^ = 91%), necessitating the utilization of a random-effects model for the analysis. Meta-regression analysis unveiled a decrease in the estimate with increasing age (Supplementary Figure S5). Age (estimate = 0.3843905, *p* = 0.034, 95% CI [0.0338157, 0.7349654]) might be the source of this observed heterogeneity (Table 3). Subgroup analysis further corroborated that age (*p* = 0.04, I^2^ = 76.60%) was sources of heterogeneity but did not compromise the stability of the results (Supplementary Table S4 and Supplementary Figure S3C). Sensitivity analysis indicated the relative stability of the study results (Figure 4D).

##### 3.4.1.5 Disappearance time of wheezing

Data from nine RCTs, encompassing 875 patients, provided insights into the disappearance time of wheezing. We detected a statistically significant difference in wheezing disappearance time between the group treated with WHD combined with azithromycin and the group receiving azithromycin alone, although the certainty of this evidence was very low (MD = −1.47, 95% CI [−1.72, −1.22], *p* < 0.00001) (Figure 3E; Table 2). Significant heterogeneity was present among the studies (*p* < 0.00001, I^2^ = 91%), necessitating the application of a random-effects model for the analysis. Meta-regression analysis demonstrated a decrease in the estimate with an increase in the course of disease (Supplementary Figure S6). Course of disease (estimate = 0.1671403, *p* = 0.037, 95% CI [0.0145114, 0.3197693]) might be the source of this observed heterogeneity (Table 3). Subgroup analysis further confirmed that course of disease (*p* = 0.02, I^2^ = 81.20%) was a source of heterogeneity, but it did not compromise the stability of the results (Supplementary Table S4D and Supplementary Figure S3D). Sensitivity analysis indicated that two studies (Wang et al., 2018; Chen and Liang, 2019) might be sources of heterogeneity. After excluding these studies, the results changed (MD = −1.42, 95% CI [−1.75, −1.10]); however, the stability of the results remained unaffected (Figure 4E).

#### 3.4.2 Secondary outcomes

##### 3.4.2.1 Length of hospital stay

Data from three RCTs provided information on the average length of hospital stay. A significant difference was observed between the group treated with WHD combined with azithromycin and the group receiving azithromycin alone (MD = −3.22, 95% CI [−4.55, −1.89], *p* < 0.00001) (Supplementary Figure S7). This result suggests a shorter hospitalization duration for patients treated with the combined therapy compared to those on azithromycin alone.

##### 3.4.2.2 T lymphocyte subsets

Eight RCTs reported data on CD3^+^ T lymphocytes, eight RCTs on CD4^+^ T lymphocytes, nine RCTs on CD8^+^ T lymphocytes, and nine RCTs on CD4+/CD8+ ratios. The meta-analysis revealed significant differences in CD3^+^ T lymphocytes (MD = 3.45, 95% CI [1.20, 5.71], *p* = 0.003), CD4^+^ T lymphocytes (MD = 3.71, 95% CI [3.04, 4.38], *p* < 0.00001), CD8^+^ T lymphocytes (MD = −4.13, 95% CI [−5.35, −2.90], *p* < 0.00001), and CD4+/CD8+ ratio (MD = 0.51, 95% CI [0.22, 0.80], *p* = 0.0006). These results indicate significant improvements in T lymphocyte subsets in the group receiving the combination of WHD and azithromycin compared to the group treated with azithromycin alone (Supplementary Figure S8).

##### 3.4.2.3 Inflammatory cytokines

Seven RCTs reported data on CRP, three RCTs on IL-4, nine RCTs on IL-6, seven RCTs on IL-8, six RCTs on IL-10, and ten RCTs on TNF-α. The meta-analysis demonstrated significant differences in CRP (MD = −3.31, 95% CI [−4.56, −2.06], *p* < 0.0001), IL-4 (MD = −7.17, 95% CI [−13.89, −0.45], *p* = 0.04), IL-6 (MD = −7.67, 95% CI [−9.57, −5.77], *p* < 0.0001), IL-8 (MD = −3.36, 95% CI [−4.30, −2.41], *p* < 0.00001), IL-10 (MD = −7.21, 95% CI [−9.78, −4.65], *p* < 0.0001), and TNF-α (MD = −9.20, 95% CI [−10.93, −7.46], *p* < 0.00001). These findings highlight significant improvements in inflammatory cytokine levels in the group receiving the combination of WHD and azithromycin compared to the group treated with azithromycin alone (Supplementary Figure S9).

##### 3.4.2.4 Adverse rate (safety)

Eight studies reported the incidence of adverse events (AEs) in both treatment groups. The meta-analysis revealed a significantly lower incidence of adverse events in the combination of WHD and azithromycin group compared to the azithromycin-alone group (RR = 0.42, 95% CI [0.27, 0.67], *p* = 0.0002). Specific adverse events in the WHD and azithromycin group included 6 cases of abdominal pain and diarrhea, 8 cases of nausea and vomiting, 1 case of allergy, 1 case of mild gastrointestinal reactions, 3 cases of rash, 1 case of dizziness, and 4 cases where specific adverse reaction types were not reported. In the control group treated with azithromycin alone, adverse events included 14 cases of abdominal pain and diarrhea, 17 cases of nausea and vomiting, 18 cases of mild gastrointestinal reactions, 2 cases of rash, 2 cases of dizziness, 1 case of allergy, 1 case of hoarseness, and 2 cases where specific adverse reactions were not recorded (Supplementary Table S5). This significant difference in adverse reaction incidence emphasizes the improved safety profile of the combination therapy involving WHD and azithromycin (*p* = 0.0002) (Supplementary Figure S10).

#### 3.4.3 Publication bias assessment

**FIGURE 5:**
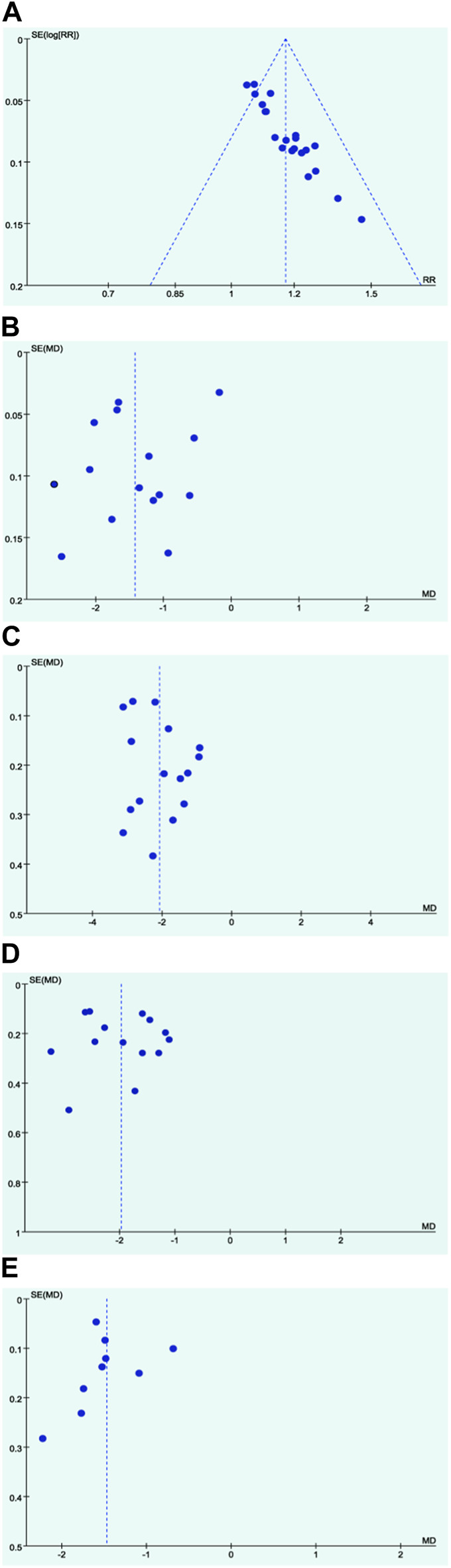
Funnel plots. **(A)** Response rate; **(B)** Disappearance time of fever; **(C)** Disappearance time of cough; **(D)** Disappearance time of pulmonary rale; **(E)** Disappearance time of wheezing.

In the assessment of potential publication bias, funnel plots were generated for key outcomes. The funnel plots for the total effective rate, fever disappearance time, cough disappearance time, and lung rales disappearance time displayed asymmetrical shapes, suggesting the possibility of publication bias (Figure 5). This observation indicates that there might be an over representation of studies with positive results in the published literature, highlighting the need for cautious interpretation of these outcomes.

## 4 Discussion

### 4.1 Main findings

This study conducted a comprehensive evaluation of the efficacy and safety of the combination therapy involving WHD and azithromycin for the treatment of MPP in pediatric patients. The analysis encompassed 22 studies and included 2026 pediatric patients. Despite the presence of biases identified within the included studies, our investigation focused on key primary outcomes, including the total effective rate, fever disappearance time, cough disappearance time, lung rales disappearance time, and wheezing disappearance time. The findings of our analysis indicated that the combination therapy of WHD and azithromycin exhibited superior efficacy in comparison to azithromycin used alone. However, it is essential to note that the certainty of these results was assessed as low. To delve into the underlying factors contributing to the heterogeneity observed in the primary outcomes, we conducted a meta-regression analysis. The results of the meta-regression highlighted that course of disease and sample size were the primary factors influencing the heterogeneity in fever disappearance time. Additionally, age emerged as the predominant factor affecting lung rales disappearance time, while course of disease played a significant role in wheezing disappearance time. It is worth noting that despite the presence of these sources of heterogeneity, they did not significantly impact the stability of our results. Moreover, in sensitivity analysis, certain studies, specifically (Wang et al., 2018; Chen and Liang, 2019; Ma and Feng, 2022), were identified as major sources of heterogeneity. However, even after excluding these studies, our results remained robust and credible.

Through a rigorous systematic study incorporating meta-regression, subgroup analysis, and sensitivity analysis, our research discovered that the combination therapy involving WHD and azithromycin exhibited statistically significant outcomes in primary measures when compared to azithromyc in administered alone. However, the quality of evidence for these primary outcomes was evaluated as low certainty or very low certainty, interpreting these results should be approached with caution. Moreover, our analysis extended to comprehensive examinations of secondary outcome measures, including the average length of hospital stay, T lymphocyte subsets (CD3^+^, CD4^+^, CD8^+^, and CD4^+^/CD8^+^ ratio), and inflammatory cytokines (CRP, IL-6, IL-8, IL-10, and TNF-α). The results of the meta-analysis for all these secondary outcomes consistently favored the combination of WHD and azithromycin over azithromycin monotherapy, and these differences were statistically significant. This not only underscores the efficacy of the WHD and azithromycin combination in primary outcome measures but also highlights its distinct advantages in secondary outcome measures.

Regarding safety, our analysis encompassed adverse events (AEs) documented in 8 RCTs, totaling 81 cases, which constituted 9.41% of the total population under study. Specifically, in the control group, 57 individuals encountered adverse reactions, constituting 13.25% of the group. In contrast, in the intervention group, 24 individuals experienced adverse reactions, accounting for 5.58%. The adverse reaction rate in the intervention group was notably lower than the overall adverse event occurrence rate and also lower than the probability of AEs in the control group. This underscores the relative safety of the treatment regimen involving the combination of WHD and azithromycin, demonstrating a comparatively lower incidence of adverse reactions.

WHD constitutes a complex traditional Chinese medicine decoction comprised of various botanical drugs and mineral components, including Ephedra sinica Stapf, Prunus armeniaca L., *Glycyrrhiza glabra* L., *Ilex pubescens* Hook. & Arn., *Pinellia ternata* (Thunb.) Makino, *Morus alba* L., and Gypsum, among others. Pharmacological studies in China have elucidated that WHD possesses the ability to alleviate airway inflammation, exert anti-allergic effects, and act as a bronchodilator by modulating the expression of proteins such as Muc5AC, STAT3, NF-κB, and NLRP3 (Dai et al., 1997; Yu et al., 2023). Ephedra sinica Stapf, a pivotal component of WHD, is widely employed globally to address symptoms like asthma, cold, flu, chills, fever, headache, nasal congestion, and cough (González-Juárez et al., 2020). *Glycyrrhiza glabra* L., another essential herb, is a perennial plant found in regions spanning southwestern Asia, the Eurasian continent, and the Mediterranean area. *Glycyrrhiza glabra* L. encompasses diverse active compounds, including glycyrrhizin and its salts, which exhibit multiple biological activities such as antiviral effects. *Glycyrrhiza glabra* L. also contains triterpene saponins, flavonoids, coumarins, and carbohydrates. Glycyrrhizin and its metabolite, glycyrrhetinic acid, have demonstrated inhibitory effects on the expression of inducible nitric oxide synthase (iNOS), thereby reducing nitric oxide production. This plays a pivotal role in the pathological mechanism of acute lung injury (Zhao et al., 2016). *Prunus armeniaca* L., the mature dried seed of a plant in the Rosaceae family, includes varieties such as bitter almonds, Siberian apricot, and northeastern apricot. The primary active component in *Prunus armeniaca* L. is amygdalin, known for its anti-asthmatic, anti-inflammatory, and antitussive properties (Wang et al., 2019; Zhang et al., 2020; Karimi et al., 2021; Wang et al., 2021). Research has indicated that the combined decoction of *Ephedra sinica* Stapf and *Prunus armeniaca* L. can enhance clinical efficacy and mitigate the toxicity of *Prunus armeniaca* L. (Qin et al., 2021). Gypsum, through its trace elements, inhibits the synthesis of cAMP and PGE2, reduces the intracellular Na/Ca ratio, enhances the action of cGMP, and suppresses the overexcitation of the body’s temperature regulation center, exerting antipyretic effects (Yang et al., 2016). Additionally, gypsum reduces the release of inflammatory mediators such as IL-6 and IFN-γ in tissue cells, alleviates inflammation by decreasing the expression of MMP-9 and MMP-2 (Jiang et al., 2022). The study (Tang et al., 2014) found that tea extract can inhibit the generation of inflammatory mediators by suppressing the TNF-α/NF-κB pathway, reducing the expression of its endogenous inflammatory factors, and exerting anti-inflammatory effects. Yu et al. (2010) discovered that tea extract can lower the levels of IgE and leukotrienes in the serum of model rats, reduce the number of eosinophils, and decrease the inflammation area in lung tissues.

Additionally, other constituents of WHD, such as *Pinellia ternata* (Thunb.) Makino and *Morus alba* L., have demonstrated remarkable effects in regulating inflammation, alleviating cough, and mitigating airway or pulmonary inflammation (Pastorino et al., 2018). These combined actions of WHD’s components contribute to its efficacy in the treatment of MPP in children.

### 4.2 Strengths

1) Widespread Clinical Application: In China, the WHD has been widely utilized by clinicians in the treatment of MPP. This study marks the first evaluation of the effectiveness and safety of the combination therapy using WHD and azithromycin for pediatric MPP. 2) Detailed Search Strategy: We conducted searches across 12 Chinese and English databases, employing meticulous and precise search strategies. Rigorous control of inclusion and exclusion criteria was maintained, minimizing potential publication bias risks. 3) Control of Heterogeneity: Considering the unique characteristics of the disease and clinical medicine, we utilized regression analysis to identify potential sources of heterogeneity, thereby reducing potential confounding factors. 4) In-Depth Analytical Approaches: We employed subgroup analysis and sensitivity analysis to thoroughly explore and elucidate potential sources of heterogeneity that could impact result stability.

### 4.3 Limitations

1) Unclear Randomization and Blinding Methods: None of the included studies reported the concealment of allocation, and seven studies did not specify their randomization methods clearly. Most studies did not mention whether blinding was employed, potentially producing bias. 2) Geographical Restriction: All included studies were limited to East Asian regions, potentially limiting the generalizability of the results to broader populations. 3) Small Sample Sizes: All studies originated from single medical centers, with many having small sample sizes. This could impact the stability and generalizability of the results. 4) Lack of Follow-Up Data: None of the included studies provided information about follow-up, potentially affecting the long-term stability assessment and safety evaluation. 5) Absence of Information on WHD’s Impact on Azithromycin Side Effects: The studies did not indicate whether WHD influenced the adverse reactions of Azithromycin, particularly concerning gastrointestinal side effects. 6) Lack of Research on Severe *Mycoplasma* Pneumoniae Pneumonia and Refractory *Mycoplasma* Pneumoniae Pneumonia, Recurrence Rates, and Economic Evaluation: Studies did not explore aspects such as the transition to severe *Mycoplasma* pneumoniae pneumonia, refractory cases, recurrence rates, and economic evaluations. 7) Many clinical outcome benefits and adverse effects are a consequence of cumulative drug action. Due to the limitations of the original study, the manuscript does not expatiate the impact of the cumulative effect of Wuhu Decoction. In the future, more relevant clinical and pharmacological studies can be carried out to explore the mechanism, dose-respones relationship and cumulative effect of Wuhu Decoction in the treatment of *mycoplasma* pneumoniae pneumonia in children.

## 5 Conclusion

Based on our meta-analysis findings, the combined therapy of Wuhu Decoction and azithromycin for treating pediatric MPP exhibited superior overall efficacy in comparison to azithromycin monotherapy. However, in the included 22 studies, the majority of evaluated factors showed unclear bias risks, and a persistent bias risk was consistently present within one category. Moreover, due to the low quality of evidence, interpreting these results should be approached with caution. In the future, pharmacological research on the treatment of MPP with WHD should be placed in a prominent position. It is hoped that through modern research methods, the mechanism of action of WHD can be elucidated more clearly. Additionally, future studies should focus more on the scientific rigor of clinical trials, and it is expected that more scientifically designed and rigorous RCTs will be conducted to further validate the efficacy of WHD.

## Data Availability

The original contributions presented in the study are included in the article/Supplementary Material, further inquiries can be directed to the corresponding author.

## Abbreviations

WHD, Wuhu Decoction; RCTs, randomized controlled trials; RR, risk ratio; CI, confidence interval; MD, mean differences; MPP, *Mycoplasma* pneumoniae pneumonia.
