# Supplementary material for: Wuhu decoction combined with azithromycin for treatment of Mycoplasma pneumoniae pneumonia in Asian children: a systematic review and meta analysis of randomized controlled trials
Source: Front Pharmacol. 2024 Apr 3;15:1329516. doi: 10.3389/fphar.2024.1329516 (PMC11021718; doi:10.3389/fphar.2024.1329516)

**Wuhu Decoction combined with azithromycin for treatment of Mycoplasma pneumoniae pneumonia in Asian children: A systematic review and meta analysis of randomized controlled trials**

**Supplementary Files**

**Contents**

Supplementary [Table 1 Information table of medicine dosage in WHD 1](#_Toc10976)

[Supplementary Table 2 PRISMA 2020 Checklist 4](#_Toc11401)

[Supplementary Table 3 Systematic search detail 8](#_Toc25806)

[Supplementary Table 4 Characteristics of included studies 10](#_Toc32129)

[Supplementary Table 5 The results of subgroup analysis 15](#_Toc10385)

[Supplementary Table 6 Details of adverse events 16](#_Toc23258)

[Supplementary Fig. 1 Regression plot of response rate 17](#_Toc128)

[Supplementary Fig. 2 Regression plot of disappearance time of fever 18](#_Toc30990)

[Supplementary Fig. 3 Forest plot of the subgroup analysis 19](#_Toc4960)

[Supplementary Fig. 4 Regression plot of disappearance time of cough 20](#_Toc9701)

[Supplementary Fig. 5 Regression plot of disappearance time of pulmonary rale 21](#_Toc16793)

[Supplementary Fig. 6 Regression plot of disappearance time of wheezing 22](#_Toc16678)

[Supplementary Fig. 7 Forest plot of the length of hospital stay 23](#_Toc16405)

[Supplementary Fig. 8 Forest plot of the T lymphocyte subsets 24](#_Toc5568)

[Supplementary Fig. 9 Forest plot of the inflammatory cytokines 25](#_Toc16713)

[Supplementary Fig. 10 Forest plot of the adverse rate (safety) 26](#_Toc238)

# Supplementary Table 1: Information table of medicine dosage in WHD

| Study | Prescription name | Ingredients of WHD (Scientific name) | Ingredients of WHD (Latin name) |
| --- | --- | --- | --- |
| Chen et al.,2019 | Wuhu Decoction | Ephedra sinica Stapf5g,Gypsum30g, Ilex pubescens Hook. & Arn. 15g,Prunus armeniaca L.10g, Morus alba L. 10g, Pinellia ternata (Thunb.) Makino10g,Glycyrrhiza glabra L. 6g | Ephedra Herba5g,Gypsum Fibrosum30g,Camellia sinensis O. Ktze.15g,Arum Ternatum Thunb.10g,Mori Cortex10g,Amygdalus Communis Vas10g,licorice6g |
| Ma et al.,2022 | Wuhu Decoction | Ephedra sinica Stapf5g,Gypsum30g,Ilex pubescens Hook.& Arn. 15g, Pinellia ternata (Thunb.) Makino10g, Morus alba L. 10g, Prunus armeniaca L. 10g, Glycyrrhiza glabra L. 6g | Ephedra Herba5g,Gypsum Fibrosum30g,Camellia sinensis O. Ktze.15g,Arum Ternatum Thunb.10g,Mori Cortex10g,Amygdalus Communis Vas10g,licorice6g |
| Deng et al.,2021 | Wuhu Decoction | Ephedra sinica Stapf3g,Gypsum10g, Pinellia ternata (Thunb.) Makino6g, Morus alba L. 10g, Prunus armeniaca L.10g, Glycyrrhiza glabra L. 6g | Ephedra Herba3g,Gypsum Fibrosum10g,Arum Ternatum Thunb.6g,Mori Cortex10g,Amygdalus Communis Vas10g,licorice6g |
| Xi et al.,2015 | Wuhu Decoction | Ephedra sinica Stapf5g,Gypsum30g, Ilex pubescens Hook. & Arn. 15g,Prunus armeniaca L.10g, Morus alba L. 10g, Pinellia ternata (Thunb.) Makino 10g,Glycyrrhiza glabra L.6g | Ephedra Herba5g,Gypsum Fibrosum30g,Camellia sinensis O. Ktze.15g,Arum Ternatum Thunb.10g,Mori Cortex10g,Amygdalus Communis Vas10g,licorice6g |
| Gong,2023 | Wuhu Decoction | Ephedra sinica Stapf3g,Gypsum5g, Pinellia ternata (Thunb.) Makino 6g, Morus alba L.5g, Prunus armeniaca L. 5g,Glycyrrhiza glabra L.6g | Ephedra Herba3g,Gypsum Fibrosum5g,Arum Ternatum Thunb.6g,Mori Cortex5g,Amygdalus Communis Vas5g,licorice6g |
| Liu,2016 | Wuhu Decoction | Ephedra sinica Stapf5g,Gypsum20g, Prunus armeniaca L.10g, Pinellia ternata (Thunb.) Makino6g,Glycyrrhiza glabra L.6g | Ephedra Herba5g,Gypsum Fibrosum20g,Amygdalus Communis Vas10g,Arum Ternatum Thunb.6g,licorice6g |
| Li et al.,2022 | Wuhu Decoction | Ephedra sinica Stapf5g,Gypsum30g, Ilex pubescens Hook. & Arn. 15g, Prunus armeniaca L. 10g, Pinellia ternata (Thunb.) Makino10g, Morus alba L. 10g,Glycyrrhiza glabra L.6g | Ephedra Herba5g,Gypsum Fibrosum30g,Camellia sinensis O. Ktze.15g,Amygdalus Communis Vas10g,Arum Ternatum Thunb.10g,Mori Cortex10g,licorice6g |
| Liu et al.,2017 | Wuhu Decoction | Ephedra sinica Stapf5g, Prunus armeniaca L. 15g,Gypsum30g,Glycyrrhiza glabra L.10g, Morus alba L.15g, Ilex pubescens Hook. & Arn. 10g, Pinellia ternata (Thunb.) Makino15g | Ephedra Herba5g,Amygdalus Communis Vas15g,Gypsum Fibrosum30g,licorice10g,Mori Cortex15g,Camellia sinensis O. Ktze.10g,Arum Ternatum Thunb.15g |
| Wang,2015 | Wuhu Decoction | Ephedra sinica Stapf5g,Gypsum15~30g, Morus alba L.6~10g, Prunus armeniaca L.6~10g, Pinellia ternata (Thunb.) Makino6~10g,Glycyrrhiza glabra L.3g | Ephedra Herba5g,Gypsum Fibrosum15~30g,Mori Cortex6~10g,Amygdalus Communis Vas6~10g,Arum Ternatum Thunb.6~10g,licorice3g |
| Qi,2021 | Wuhu Decoction | Ephedra sinica Stapf3g,Gypsum18g, Prunus armeniaca L.6g, Morus alba L.6g,Glycyrrhiza glabra L.3g | Ephedra Herba3g,Gypsum Fibrosum18g,Amygdalus Communis Vas6g,Mori Cortex6g,licorice3g |
| Wang et al.,2018 | Wuhu Decoction | Ephedra sinica Stapf5g, Prunus armeniaca L.10g,Gypsum30g,Glycyrrhiza glabra L.6g, Morus alba L. 10g, Pinellia ternata (Thunb.) Makino10g,Ilex pubescens Hook.& Arn. 15g | Ephedra Herba5g,Amygdalus Communis Vas10g,Gypsum Fibrosum30g,licorice6g,Mori Cortex10g,Arum Ternatum Thunb.10g,Camellia sinensis O. Ktze.15g |
| Shi et al.,2022 | Wuhu Decoction | Ephedra sinica Stapf3g, Pinellia ternata (Thunb.) Makino6g, Prunus armeniaca L. 10g,Gypsum10g,Morus alba L. 10g,Glycyrrhiza glabra L.6g | Ephedra Herba3g,Arum Ternatum Thunb.6g,Amygdalus Communis Vas10g,Gypsum Fibrosum10g,Mori Cortex10g,licorice6g |
| Su,2016 | Wuhu Decoction | Ephedra sinica Stapf6g,Gypsum30g, Prunus armeniaca L.12g,Morus alba L. 12g, Pinellia ternata (Thunb.) Makino12g,Ilex pubescens Hook.& Arn. 15g,Glycyrrhiza glabra L.6g | Ephedra Herba6g,Gypsum Fibrosum30g,Amygdalus Communis Vas12g,Mori Cortex12g,Arum Ternatum Thunb.12g,Camellia sinensis O. Ktze.15g,licorice6g |
| Li et al.,2020 | Wuhu Decoction | Ephedra sinica Stapf5g, Prunus armeniaca L.10g,Glycyrrhiza glabra L.6g,Morus alba L. 10g | Ephedra Herba5g,Amygdalus Communis Vas10g,licorice6g,Mori Cortex10g |
| Ren et al.,2020 | Wuhu Decoction | Ephedra sinica Stapf5g, Prunus armeniaca L.10g,Gypsum30g,Glycyrrhiza glabra L.6g,Morus alba L. 10g, Ilex pubescens Hook. & Arn. 15g, Pinellia ternata (Thunb.) Makino10g | Ephedra Herba5g,Amygdalus Communis Vas10g,Gypsum Fibrosum30g,licorice6g,Mori Cortex10g,Camellia sinensis O. Ktze.15g,Arum Ternatum Thunb.10g |
| Qi,2019 | Wuhu Decoction | Ephedra sinica Stapf10g, Prunus armeniaca L.10g, Pinellia ternata (Thunb.) Makino5g,Glycyrrhiza glabra L.10g,Gypsum30g | Ephedra Herba10g,Amygdalus Communis Vas10g,Arum Ternatum Thunb.5g,licorice10g,Gypsum Fibrosum30g |
| Chen,2019 | Wuhu Decoction | Ephedra sinica Stapf3-5g,Gypsum30g, Prunus armeniaca L.3-6g,Glycyrrhiza glabra L.3g, Morus alba L.5-10g | Gypsum Fibrosum30g,Amygdalus Communis Vas3-6g,licorice3g,Ephedra Herba3-5g,Mori Cortex5-10g |
| Yan,2019 | Wuhu Decoction | Ephedra sinica Stapf5g,Gypsum20g, Prunus armeniaca L.10g,Glycyrrhiza glabra L.6g, Pinellia ternata (Thunb.) Makino6g | Gypsum Fibrosum20g,Amygdalus Communis Vas10g,licorice6g,Arum Ternatum Thunb.6g,Ephedra Herba5g |
| Bian et al.,2018 | Wuhu Decoction | Prunus armeniaca L.12g, Pinellia ternata (Thunb.) Makino12g,Morus alba L. 12g,Ilex pubescens Hook.& Arn. 15g,Gypsum30g,Ephedra sinica Stapf6g,Glycyrrhiza glabra L.6g | Amygdalus Communis Vas12g,Arum Ternatum Thunb.12g,Mori Cortex12g,Camellia sinensis O. Ktze.15g,Gypsum Fibrosum30g,Ephedra Herba6g,licorice6g |
| Yang,2023 | Wuhu Decoction | Ephedra sinica Stapf5g,Glycyrrhiza glabra L.6g, Prunus armeniaca L.10g,Morus alba L. 10g,Ilex pubescens Hook.& Arn. 15 g,Gypsum25g | Ephedra Herba5g,licorice6g,Amygdalus Communis Vas10g,Mori Cortex10g,Camellia sinensis O. Ktze.15g,Gypsum Fibrosum25g |
| Wu et al.,2018 | Wuhu Decoction | Ephedra sinica Stapf3-5g, Prunus armeniaca L.6-9g, Pinellia ternata (Thunb.) Makino6-9g,Morus alba L. 8-10g,Ilex pubescens Hook.& Arn. 10-15g,Gypsum15-30g,Glycyrrhiza glabra L.3-6g | Ephedra Herba3-5g,Amygdalus Communis Vas6-9g,Arum Ternatum Thunb.6-9g,Mori Cortex8-10g,Camellia sinensis O. Ktze.10-15g,Gypsum Fibrosum15-30g,licorice3-6g |
| Dong et al.,2018 | Wuhu Decoction | Ephedra sinica Stapf3g,Gypsum20g, Prunus armeniaca L.8g,Morus alba L. 8g, Pinellia ternata (Thunb.) Makino10g,Glycyrrhiza glabra L.3g | Ephedra Herba3g,Gypsum Fibrosum20g,Amygdalus Communis Vas8g,Mori Cortex8g,Arum Ternatum Thunb.10g,licorice3g |

# Supplementary Table 2: PRISMA 2020 Checklist

| **Section and Topic** | **Item #** | **Checklist item** | **Location where item is reported** |
| --- | --- | --- | --- |
| **TITLE** | | |  |
| Title | 1 | Identify the report as a systematic review,meta-analysis, or both. |  |
| **ABSTRACT** | | |  |
| Abstract | 2 | Provide a structured summary, including: study purpose, data source, research method, study content, study time limit, interventions, study results, results analysis, conclusions, limitations. |  |
| **INTRODUCTION** | | |  |
| Rationale | 3 | Describe the rationale for the review in the context of existing knowledge. |  |
| Objectives | 4 | Provide an explicit statement of questions being addressed with reference to participants, interventions, comparisons, outcomes, and study design (PICOS). |  |
| **METHODS** | | |  |
| Eligibility criteria | 5 | Specify the inclusion and exclusion criteria for the review and how studies were grouped for the syntheses. |  |
| Information sources | 6 | Specify all databases, registers, websites, organisations, reference lists and other sources searched or consulted to identify studies. Specify the date when each source was last searched or consulted. |  |
| Search strategy | 7 | Present the full search strategies for all databases, registers and websites, including any filters and limits used. | supplementary table 2 |
| Selection process | 8 | Specify the methods used to decide whether a study met the inclusion criteria of the review, including how many reviewers screened each record and each report retrieved, whether they worked independently, and if applicable, details of automation tools used in the process. |  |
| Data collection process | 9 | Specify the methods used to collect data from reports, including how many reviewers collected data from each report, whether they worked independently, any processes for obtaining or confirming data from study investigators, and if applicable, details of automation tools used in the process. |  |
| Data items | 10a | List and define all outcomes for which data were sought. Specify whether all results that were compatible with each outcome domain in each study were sought (e.g. for all measures, time points, analyses), and if not, the methods used to decide which results to collect. |  |
|  | 10b | List and define all other variables for which data were sought (e.g. participant and intervention characteristics, funding sources). Describe any assumptions made about any missing or unclear information. |  |
| Study risk of bias assessment | 11 | Specify the methods used to assess risk of bias in the included studies, including details of the tool(s) used, how many reviewers assessed each study and whether they worked independently, and if applicable, details of automation tools used in the process. |  |
| Effect measures | 12 | Specify for each outcome the effect measure(s) (e.g. risk ratio, mean difference) used in the synthesis or presentation of results. |  |
| Synthesis methods | 13a | Describe the processes used to decide which studies were eligible for each synthesis (e.g. tabulating the study intervention characteristics and comparing against the planned groups for each synthesis (item #5)). |  |
|  | 13b | Describe any methods required to prepare the data for presentation or synthesis, such as handling of missing summary statistics, or data conversions. |  |
|  | 13c | Describe any methods used to tabulate or visually display results of individual studies and syntheses. |  |
|  | 13d | Describe any methods used to synthesize results and provide a rationale for the choice(s). If meta-analysis was performed, describe the model(s), method(s) to identify the presence and extent of statistical heterogeneity, and software package(s) used. |  |
|  | 13e | Describe any methods used to explore possible causes of heterogeneity among study results (e.g. subgroup analysis, meta-regression). |  |
|  | 13f | Describe any sensitivity analyses conducted to assess robustness of the synthesized results. |  |
| Reporting bias assessment | 14 | Describe any methods used to assess risk of bias due to missing results in a synthesis (arising from reporting biases). |  |
| Certainty assessment | 15 | Describe any methods used to assess certainty (or confidence) in the body of evidence for an outcome. |  |
| **RESULTS** | | |  |
| Study selection | 16a | Describe the results of the search and selection process, from the number of records identified in the search to the number of studies included in the review, ideally using a flow diagram. | Figure 1 |
|  | 16b | Cite studies that might appear to meet the inclusion criteria, but which were excluded, and explain why they were excluded. |  |
| Study characteristics | 17 | Cite each included study and present its characteristics. | supplementary table 3 |
| Risk of bias in studies | 18 | Present assessments of risk of bias for each included study. | Figure 2 |
| Results of individual studies | 19 | For all outcomes, present, for each study: (a) summary statistics for each group (where appropriate) and (b) an effect estimate and its precision (e.g. confidence/credible interval), ideally using structured tables or plots. | Figure 3 |
| Results of syntheses | 20a | For each synthesis, briefly summarise the characteristics and risk of bias among contributing studies. |  |
|  | 20b | Present results of all statistical syntheses conducted. If meta-analysis was done, present for each the summary estimate and its precision (e.g. confidence/credible interval) and measures of statistical heterogeneity. If comparing groups, describe the direction of the effect. |  |
|  | 20c | Present results of all investigations of possible causes of heterogeneity among study results. |  |
|  | 20d | Present results of all sensitivity analyses conducted to assess the robustness of the synthesized results. | Figure 4 |
| Reporting biases | 21 | Present assessments of risk of bias due to missing results (arising from reporting biases) for each synthesis assessed. | Figure 5 |
| Certainty of evidence | 22 | Present assessments of certainty (or confidence) in the body of evidence for each outcome assessed. | Table 2 |
| **DISCUSSION** | | |  |
| Discussion | 23a | Provide a general interpretation of the results in the context of other evidence. |  |
|  | 23b | Discuss any limitations of the evidence included in the review. |  |
|  | 23c | Discuss any limitations of the review processes used. |  |
|  | 23d | Discuss implications of the results for practice, policy, and future research. |  |
| **OTHER INFORMATION** | | |  |
| Registration and protocol | 24a | Provide registration information for the review, including register name and registration number, or state that the review was not registered. |  |
|  | 24b | Indicate where the review protocol can be accessed, or state that a protocol was not prepared. |  |
|  | 24c | Describe and explain any amendments to information provided at registration or in the protocol. |  |
| Support | 25 | Describe sources of financial or non-financial support for the review, and the role of the funders or sponsors in the review. |  |
| Competing interests | 26 | Declare any competing interests of review authors. |  |
| Availability of data, code and other materials | 27 | Report which of the following are publicly available and where they can be found: template data collection forms; data extracted from included studies; data used for all analyses; analytic code; any other materials used in the review. |  |

# Supplementary Table 3 Systematic search detail

| **The PubMed database Search Strategy** | | |
| --- | --- | --- |
| **Sequence** | **Search terms** | **Hits** |
| #1 | (((((Mycoplasma pneumoniae pneumonia[MeSH Terms]) OR (Mycoplasma pneumoniae pneumonia in children[Title/Abstract])) OR (MPP[Title/Abstract])) OR (Mycoplasma pneumonia[Title/Abstract])) OR (Mycoplasma pneumonia in children[Title/Abstract])) OR (Mycoplasma pneumoniae in pediatric patients[Title/Abstract]) | 10549 |
| #2 | ((Wuhu Decoction[Title/Abstract]) OR (WHD[Title/Abstract])) OR (Wuhu Tang[Title/Abstract]) | 138 |
| #3 | (((((((((randomized controlled study[Title/Abstract]) OR randomized controlled trial[Title/Abstract]) OR randomized trial[Title/Abstract]) OR randomized study[Title/Abstract]) OR randomized placebo-controlled study[Title/Abstract]) OR randomized parallel-group study[Title/Abstract]) OR controlled clinical trial[Title/Abstract]) OR multicenter study[Title/Abstract]) OR double-blinded controlled study[Title/Abstract]) | 253100 |
| #4 | #1 AND #2 AND #3 | 0 |
| **The Web of science database Search Strategy** | | |
| **Sequence** | **Search terms** | **Hits** |
| #1 | **(((((TS=(Mycoplasma pneumoniae pneumonia)) OR TS=(Mycoplasma pneumoniae pneumonia in children)) OR TS=(MPP)) OR TS=(Mycoplasma pneumonia)) OR TS=(Mycoplasma pneumonia in children)) OR TS=(Mycoplasma pneumoniae in pediatric patients)** | 14122 |
| #2 | **((TS=(**Wuhu Decoction**)) OR TS=(WHD)) OR TS=(**Wuhu Tang**)** | 134 |
| #3 | **((((((((TS=(randomized controlled study)) OR TS=(randomized controlled trial)) OR TS=(randomized trial)) OR TS=(randomized study)) OR TS=(randomized placebo-controlled study)) OR TS=(randomized parallel-group study)) OR TS=(controlled clinical trial)) OR TS=( multicenter study)) OR TS=(double-blinded controlled study)** | 1262022 |
| #4 | #1 AND #2 AND #3 | 26,801 |
| **The Cochrane library database Search Strategy** | | |
| **Sequence** | **Search terms** | **Hits** |
| #1 | (Mycoplasma pneumoniae pneumonia or Mycoplasma pneumoniae pneumonia in children or MPP or Mycoplasma pneumonia or Mycoplasma pneumonia in children or Mycoplasma pneumoniae in pediatric patients):ti,ab,kw | 535 |
| #2 | (Wuhu Decoction or WHD or Wuhu Tang):ti,ab,kw | 11 |
| #3 | (randomized controlled study or randomized controlled trial or randomized trial or randomized study or randomized placebo-controlled study or randomized parallel-group study or controlled clinical trial or multicenter study or double-blinded controlled study):ti,ab,kw | 1389339 |
| #4 | #1 AND #2 AND #3 | 0 |
| **The Embase database Search Strategy** | | |
| **Sequence** | **Search terms** | **Hits** |
| #1 | 'mycoplasma pneumoniae pneumonia':ab,ti OR 'mycoplasma pneumoniae pneumonia in children':ab,ti OR 'MPP':ab,ti OR 'mycoplasma pneumonia':ab,ti OR 'mycoplasma pneumonia in children':ab,ti OR 'mycoplasma pneumoniae in pediatric patients':ab,ti | 9174 |
| #2 | 'Wuhu Decoction':ab,ti OR 'WHD':ab,ti OR 'Wuhu Tang':ab,ti | 152 |
| #3 | 'randomized controlled study':ab,ti OR 'randomized controlled trial':ab,ti OR 'randomized trial':ab,ti OR 'randomized study':ab,ti OR 'randomized placebo-controlled study':ab,ti OR 'randomized parallel-group study':ab,ti OR 'controlled clinical trial':ab,ti OR 'multicenter study':ab,ti OR 'double-blinded controlled study':ab,ti | 339822 |
| #4 | #1 AND #2 AND #3 | 0 |
| **The Ovid database Search Strategy** | | |
| **Sequence** | **Search terms** | **Hits** |
| #1 | (Mycoplasma pneumoniae pneumonia or Mycoplasma pneumoniae pneumonia in children or MPP or Mycoplasma pneumonia or Mycoplasma pneumonia in children or Mycoplasma pneumoniae in pediatric patients).ab. | 1051 |
| #2 | (Wuhu Decoction or WHD or Wuhu Tang).ab. | 23 |
| #3 | (randomized controlled study or randomized controlled trial or randomized trial or randomized study or randomized placebo-controlled study or randomized parallel-group study or controlled clinical trial or multicenter study or double-blinded controlled study).ab. | 59510 |
| #4 | #1 AND #2 AND #3 | 0 |
| **The MEDILINE database Search Strategy** | | |
| **Sequence** | **Search terms** | **Hits** |
| #1 | SU mycoplasma pneumoniae pneumonia OR mycoplasma pneumoniae pneumonia in children OR MPP OR mycoplasma pneumonia OR mycoplasma pneumonia in children OR mycoplasma pneumoniae in pediatric patients | 2388 |
| #2 | SU Wuhu Decoction OR WHD OR Wuhu Tang | 5 |
| #3 | SU randomized controlled study OR randomized controlled trial OR randomized trial OR randomized study OR randomized placebo-controlled study OR randomized parallel-group study OR controlled clinical trial OR multicenter study OR double-blinded controlled study | 343,241 |
| #4 | #1 AND #2 AND #3 | 0 |
| **Results of the references of included studies and reviews and Register the website of clinical trials** | | |
| **Retrieve the object** | | **Results** |
| References of included studied | | 0 |
| References of reviews | | 0 |
| Chinese Clinical Trial Registry (http://www.chictr.org.cn) | | 0 |

# Supplementary Table 4 Characteristics of included studies

| **Studies** | **Cases** | | **Dosage** | | **Sex (M/F)** | | **Intervention** | | **Age (years)** | | **Course of disease** | | **Course (day)** | **Outcome index** |
| --- | --- | --- | --- | --- | --- | --- | --- | --- | --- | --- | --- | --- | --- | --- |
|  | **T** | **C** | **T** | **C** | **T** | **C** | **T** | **C** | **T** | **C** | **T** | **C** |  |  |
| Chen et al., 2019 | 43 | 43 | Wuhu Decoction (3age:40ml,po,tid; 4age:50ml,po,tid;  ≥5age:80ml,po,tid)+ Azithromycin (10mg/(kg·d),po,qd) | Azithromycin (10mg/(kg·d),po,qd) | 25/18 | 27/16 | WHD+AZM+CT+AA | AZM+CT+AA | 6.01  ±  1.96 | 6.98±  2.14 | NA | NA | 14 | abcdeg |
| Ma et al., 2022 | 42 | 42 | WHD(  ≤3age:40ml,po,tid; 4-5age:50ml,po,tid;  ≥6age:80ml,po,tid)+ Azithromycin (sequential therapy:10mg/(kg·d),ivgtt,qd and 10mg/(kg·d),po,qd) | Azithromycin (sequential therapy:10mg/(kg·d),ivgtt,qd and 10mg/(kg·d),po,qd) | 25/17 | 24/18 | WHD+AZM+CT | AZM+CT | 6.12 ± 1.17 | 6.09 ± 1.22 | 7.44 ± 1.36 | 7.42 ± 1.37 | 14 | acdfijl |
| Deng et al.,2021 | 45 | 45 | WHD(  <5age:50ml,po,bid; 5-12age:100ml,po,bid)+Azithromycin(sequential therapy:10mg/(kg·d),ivgtt,qd and 10mg/(kg·d),po,qd) | Azithromycin (sequential therapy:10mg/(kg·d),ivgtt,qd and 10mg/(kg·d),po,qd) | 25/20 | 24/21 | WHD+AZM+CT | AZM+CT | 7.34 ± 1.12 | 7.35 ± 1.11 | 6.21 ± 1.31 | 6.13 ± 1.34 | 14 | acdejklmnopqrst |
| Xi et al.,2015 | 46 | 46 | WHD (2-3age:40ml,po,tid; 3-4age:50ml,po,tid;  >4age:80ml,po,tid)+ Azithromycin (10mg/(kg·d),po,qd) | Azithromycin (10mg/(kg·d),po,qd) | 31/15 | 15/19 | WHD+AZM+CT | AZM+CT | NA | NA | NA | NA | 14 | abcdeg |
| Gong,2023 | 40 | 40 | WHD(NA)+ Azithromycin (sequential therapy:10mg/(kg·d),ivgtt,qd and 10mg/(kg·d),po,qd) | Azithromycin (sequential therapy:10mg/(kg·d),ivgtt,qd and 10mg/(kg·d),po,qd) | 27/13 | 25/15 | WHD+AZM+CT | AZM+CT | 4.86  ±  0.71 | 4.91±  0.73 | 8.02±  1.01 | 7.88±  1.06 | 14 | ailm |
| Liu,2016 | 48 | 48 | WHD(50ml,po,bid;  33ml,po,tid)+ Azithromycin (sequential therapy:10mg/(kg·d),ivgtt,qd and 10mg/(kg·d),po,qd) | Azithromycin (sequential therapy:10mg/(kg·d),ivgtt,qd and 10mg/(kg·d),po,qd) | 22/26 | 23/25 | WHD+AZM+CT | AZM+CT | 5.9  ±  1.2 | 5.8±  1.3 | 4.9  ±  0.8 | 5.8  ±  1.3 | 21 | akqrst |
| Li et al.,2022 | 40 | 40 | WHD (80ml,po,tid)+ Azithromycin (10mg/(kg·d),po,qd) | Azithromycin (10mg/(kg·d),po,qd) | 20/20 | 18/22 | WHD+AZM+CT+AA | AZM+CT+AA | 7.47  ±  1.06 | 7.15±  1.31 | 9.25±  1.86 | 9.72±  2.24 | 7 | abcdehl |
| Liu et al.,2017 | 38 | 38 | WHD (2-3age:40ml,po,tid; 3-4age:50ml,po,tid;  >4age:80ml,po,tid)+ Azithromycin (10mg/(kg·d),po,qd) | Azithromycin (10mg/(kg·d),po,qd) | 19/19 | 21/17 | WHD+AZM+CT | AZM+CT | 6.33 ± 0.90 | 6.38 ± 0.91 | 6.33±  0.90 | 6.27±  0.87 | 14 | acdefhjlnopqrst |
| Wang,2015 | 32 | 32 | WHD (<3age:50-80ml,po,tid; 3-6age:80-100ml,po,tid;  >6age:100-150ml,po,tid)+ Azithromycin (10mg/(kg·d),po,qd) | Azithromycin (10mg/(kg·d),po,qd) | NA | NA | WHD+AZM+CT | AZM+CT | NA | NA | 8.91±1.27 | | NA | ab |
| Qi,2021 | 40 | 40 | WHDG(NA)+ Azithromycin (sequential therapy:10mg/(kg·d),ivgtt,qd) | Azithromycin (sequential therapy:10mg/(kg·d),ivgtt,qd) | 26/14 | 25/15 | WHDG+AZM+CT | AZM+CT | 6.24  ±  1.12 | 6.19±  1.05 | 1.97±  0.25 | 2.01±  0.23 | 10 | acdefikm |
| Wang et al.,2018 | 67 | 68 | WHD  (2-3age:40ml,po,tid; 3-4age:50ml,po,tid;  >4age:80ml,po,tid)+ Azithromycin (sequential therapy:10mg/(kg·d),ivgtt,qd and 10mg/(kg·d),po,qd) | Azithromycin (sequential therapy:10mg/(kg·d),ivgtt,qd and 10mg/(kg·d),po,qd) | 38/29 | 39/29 | WHD+AZM+CT+SHLG | AZM+CT+SHLG | 7.4  ±  2.5 | 7.5 ± 2.7 | NA | NA | 14 | acdefhijkl |
| Shi et al.,2022 | 35 | 35 | WHD(NA)+ Azithromycin (sequential therapy:10mg/(kg·d),ivgtt,qd and 10mg/(kg·d),po,qd) | Azithromycin (sequential therapy:10mg/(kg·d),ivgtt,qd and 10mg/(kg·d),po,qd) | 20/15 | 21/14 | WHD+AZM+CT | AZM+CT | 7.27  ±  2.06 | 7.31±  2.12 | 6.25±  1.21 | 6.27±  1.18 | 14 | aiklmqrst |
| Su,2016 | 57 | 57 | WHD  (80ml,po,tid)+ Azithromycin (sequential therapy:10mg/(kg·d),ivgtt,qd and 10mg/(kg·d),po,qd) | Azithromycin (sequential therapy:10mg/(kg·d),ivgtt,qd and 10mg/(kg·d),po,qd) | 25/32 | 27/30 | WHD+AZM | AZM | 8.53  ± 1.42 | 8.49 ± 1.28 | NA | NA | 11 | adijlm |
| Li et al.,2020 | 68 | 67 | WHD  (100ml,po,bid)+ Azithromycin (sequential therapy:10mg/(kg·d),ivgtt,qd and 10mg/(kg·d),po,qd) | Azithromycin (sequential therapy:10mg/(kg·d),ivgtt,qd and 10mg/(kg·d),po,qd) | 35/32 | 32/27 | WHD+AZM+CT | AZM+CT | 4.21 ± 0.43 | 4.23 ± 0.45 | 3.18±  0.32 | 3.33±  0.27 | 16 | acdejklmqrst |
| Ren et al.,2020 | 30 | 30 | WHD  (2-3age:40ml,po,tid; 3-4age:50ml,po,tid;  ≥4age:80ml,po,tid)+ Azithromycin (sequential therapy:10mg/(kg·d),ivgtt,qd and 10mg/(kg·d),po,qd) | Azithromycin (sequential therapy:10mg/(kg·d),ivgtt,qd and 10mg/(kg·d),po,qd) | 18/12 | 13/17 | WHD+AZM+CT | AZM+CT | 5.98 ± 1.35 | 6.45 ± 1.62 | 6.17±  1.52 | 6.08±  1.36 | 14 | acdefm |
| Qi,2019 | 41 | 41 | WHD(50ml,po,bid;  33ml,po,tid)+ Azithromycin (10mg/(kg·d),po,qd) | Azithromycin (10mg/(kg·d),po,qd) | 22/19 | 23/18 | WHD+AZM+CT | AZM+CT | 6.25  ±  1.46 | 6.19±  1.33 | 6.25±  1.46 | 6.19±  1.33 | 7 | acdef |
| Chen,2019 | 61 | 61 | WHD  (3-4age:50ml,po,tid; 5-7age:60ml,po,tid;  >7age:80ml,po,tid)+ Azithromycin (sequential therapy:10mg/(kg·d),ivgtt,qd and 10mg/(kg·d),po,qd) | Azithromycin (sequential therapy:10mg/(kg·d),ivgtt,qd and 10mg/(kg·d),po,qd) | 36/25 | 34/27 | WHD+AZM+CT | AZM+CT | 7.04  ±  4.22 | 7.16±  4.18 | 1.75±  1.39 | 1.65±  1.44 | 14 | acdef |
| Yan,2019 | 45 | 45 | WHD(500ml,po,bid;  330ml,po,tid)+ Azithromycin (sequential therapy:10mg/(kg·d),ivgtt,qd) | Azithromycin (sequential therapy:10mg/(kg·d),ivgtt,qd) | 24/21 | 25/20 | WHD+AZM+CT | AZM+CT | 4.11  ±  1.12 | 4.24±  0.46 | 3.19±  0.34 | 3.23±  0.28 | 21 | aqrst |
| Bian et al.,2018 | 75 | 75 | WHD  (2-3age:40ml,po,tid; 3-4age:50ml,po,tid;  >4age:80ml,po,tid)+ Azithromycin (sequential therapy:10mg/(kg·d),ivgtt,qd and 10mg/(kg·d),po,qd) | Azithromycin (sequential therapy:10mg/(kg·d),ivgtt,qd and 10mg/(kg·d),po,qd) | 42/33 | 45/30 | WHD+AZM+CT | AZM+CT | 6.07 ± 1.24 | 5.95 ± 1.06 | 6.37±  1.24 | 6.12±  1.58 | 14 | acdefijlqrst |
| Yang,2023 | 32 | 32 | WHD  (3-4age:50ml,po,tid; 4-5age:60ml,po,tid;  >5age:75ml,po,tid)+ Azithromycin (10mg/(kg·d),po,qd) | Azithromycin (10mg/(kg·d),po,qd) | 17/15 | 18/14 | WHD+AZM+CT | AZM+CT | 6.59  ±  1.62 | 6.63±  1.45 | NA | NA | NA | abiqrst |
| Wu et al.,2018 | 45 | 45 | WHD  (po,tid)+ Azithromycin (10mg/(kg·d),po,qd) | Azithromycin (10mg/(kg·d),po,qd) | 22/23 | 20/25 | WHD+AZM+CT+AA | AZM+CT+AA | 6.25  ±  2.36 | 5.69±  2.11 | 5.12 ±  1.03 | 5.28 ±  1.14 | 14 | acdegqrst |
| Dong et al.,2018 | 43 | 43 | WHD  (po,bid)+ Azithromycin (sequential therapy:10mg/(kg·d),ivgtt,qd and 10mg/(kg·d),po,qd) | Azithromycin (sequential therapy:10mg/(kg·d),ivgtt,qd and 10mg/(kg·d),po,qd) | 22/21 | 27/16 | WHD+AZM+CT | AZM+CT | 6.10  ±  2.22 | 6.15±  2.09 | 6.56±  2.40 | 6.49±  2.22 | 14 | acdefnop |

**Outcome indexes:**a:total efficiency;b:TCM Symptom Score;c:Disappearance time of fever;d:Disappearance time of cough;e:Disappearance time of pulmonary rale;f:Disappearance time of wheezing;g:hospital day;h:IL-4;i:IL-6;j:IL-8;k:IL-10;l:TNF-α;m:CRP;n:IgA;o:IgG;p:IgM;q:CD3+;r:CD4+;s:CD8+;t:CD4+/CD8+;

**Abbreviations:** T:experimental group; C:control group; WHD:Wuhu Decoction; WHDG:Wuhu Decoction Granules; M:Male; F:Female; AZM:Azithromycin; CT:Conventional Therapy;SHLG:Shuanghuanglian Granules;AA: Acupoint Application.

# Supplementary Table 5 The results of subgroup analysis

| **Outcomes** | **Category** | **P** | **I^2^** |
| --- | --- | --- | --- |
| Disappearance time of fever | Sample size | 0.0009 | 85.70% |
|  | course of disease | 0.01 | 83.20% |
| Disappearance time of pulmonary rale | Age | 0.04 | 76.60% |
| Disappearance time of wheezing | course of disease | 0.02 | 81.20% |

# Supplementary Table 6 Details of adverse events

| **ID** | **Intervention（T/C）** | | **adverse events (T)** | **Sample Size (T)** | **adverse events (C)** | **Sample Size (C)** | **abdominal pain and diarrhea (T/C)** | **nausea and vomiting (T/C)** | **Unspecified gastrointestinal reactions (T/C)** | **Skin rash (T/C)** | **Dizziness**  **(T/C)** | **Unspecified reactions**  **(T/C)** | **Allergy**  **(T/C)** | **Hoarseness**  **(T/C)** |
| --- | --- | --- | --- | --- | --- | --- | --- | --- | --- | --- | --- | --- | --- | --- |
| Ma et al., 2022 | WHD+AZM+CT | AZM+CT | 2 | 42 | 8 | 42 | 0/2 | 1/3 | 0/2 | 1/1 | / | / | / | / |
| Deng et al.,2021 | WHD+AZM+CT | AZM+CT | 4 | 45 | 8 | 45 | 2/3 | 1/2 | / | 0/1 | 1/2 | / | / | / |
| Gong,2023 | WHD+AZM+CT | AZM+CT | 4 | 40 | 2 | 40 | / | / | / | / | / | 4/2 | / | / |
| Liu,2016 | WHD+AZM+CT | AZM+CT | 3 | 48 | 10 | 48 | 1/0 | 2/0 | 0/10 | / | / | / | / | / |
| Wang et al.,2018 | WHD+AZM+CT+SHLG | AZM+CT+SHLG | 3 | 67 | 4 | 68 | 2/0 | 1/0 | 0/4 | / | / | / | / | / |
| Li et al.,2020 | WHD+AZM+CT | AZM+CT | 3 | 68 | 12 | 67 | 0/2 | 2/8 | / | / | / | / | 1/1 | 0/1 |
| Bian et al.,2018 | WHD+AZM+CT | AZM+CT | 2 | 75 | 11 | 75 | 1/7 | 1/4 | / | / | / | / | / | / |
| Wu et al.,2018 | WHD+AZM+CT+CMS | AZM+CT+CMS | 3 | 45 | 2 | 45 | / | / | 1/2 | 2/0 | / | / | / | / |

# Groups: T: Treatment group; C: Control group

# Interventions:WHD:Wuhu Decoction;AZM:Azithromycin; CT:Conventional Therapy;SHLG,Shuanghuanglian Granules;CMS,Chinese Medical Skills(such as acupoint application)

# **Supplementary Fig. 1 Regression plot of response rate**

(A)Age;(B)Dosage;(C)Sample size;(D)Regional disparities between North and South China;(E)Course of disease;(F)The use of acupoint application.**
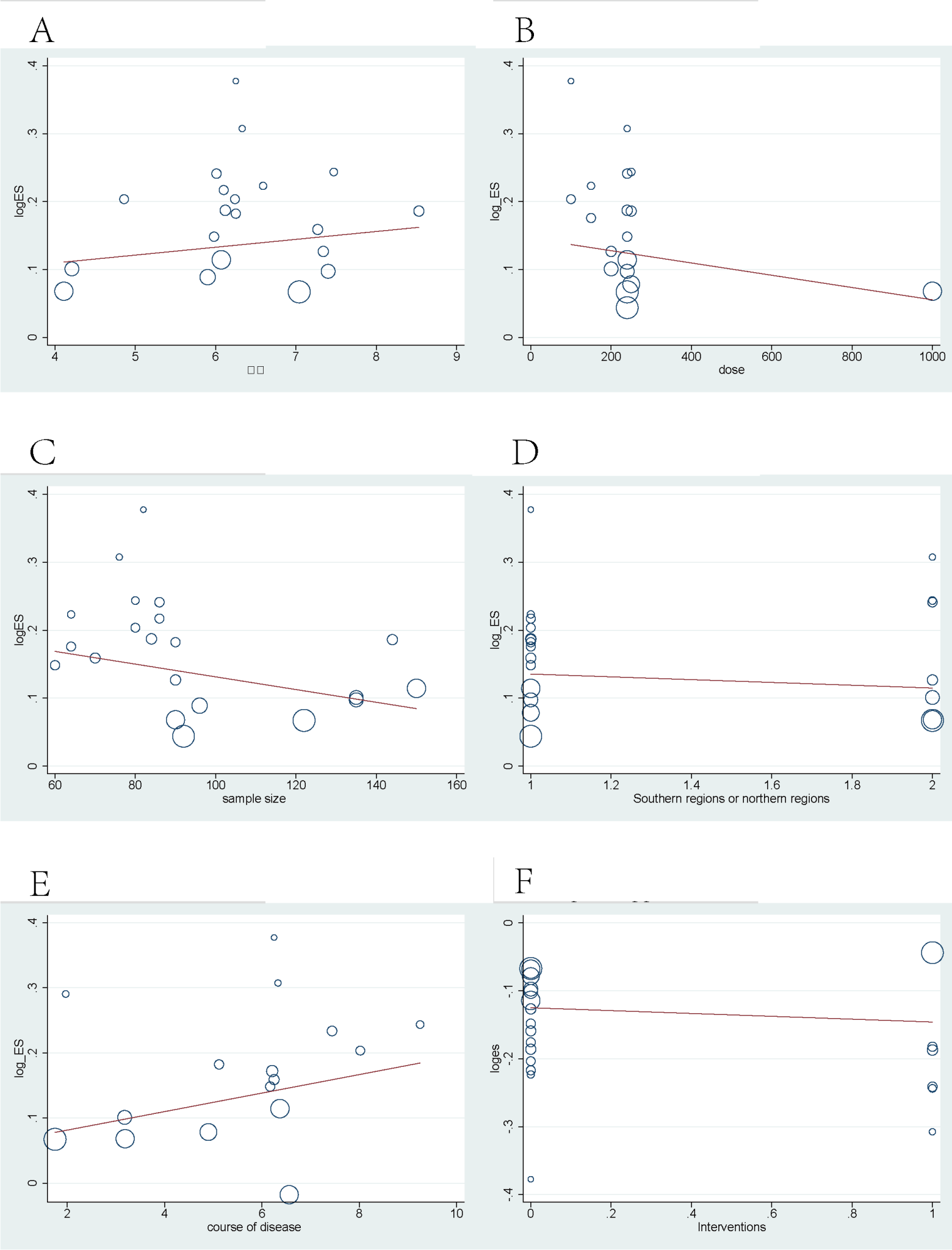
**

# **Supplementary Fig. 2 Regression plot of disappearance time of fever**

(A)Age;(B)Dosage;(C)Sample size;(D)Regional disparities between North and South China;(E)Course of disease;(F)The use of acupoint application.


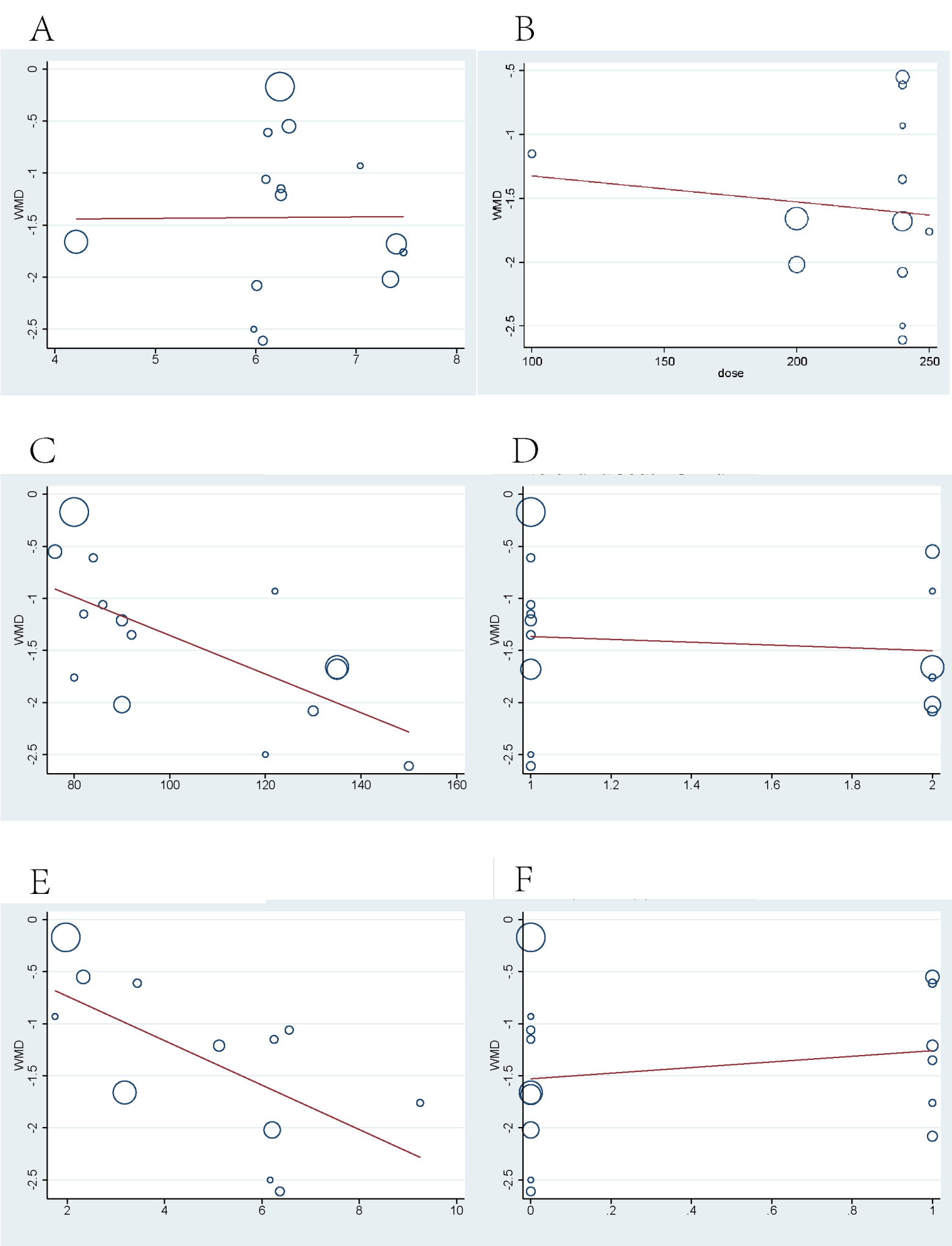


# **Supplementary Fig. 3** Forest plot of the subgroup analysis

(A) Disappearance time of fever(Sample size); (B) Disappearance time of fever(Course of disease);(C)Disappearance time of pulmonary rale; (D) Disappearance time of wheezing.


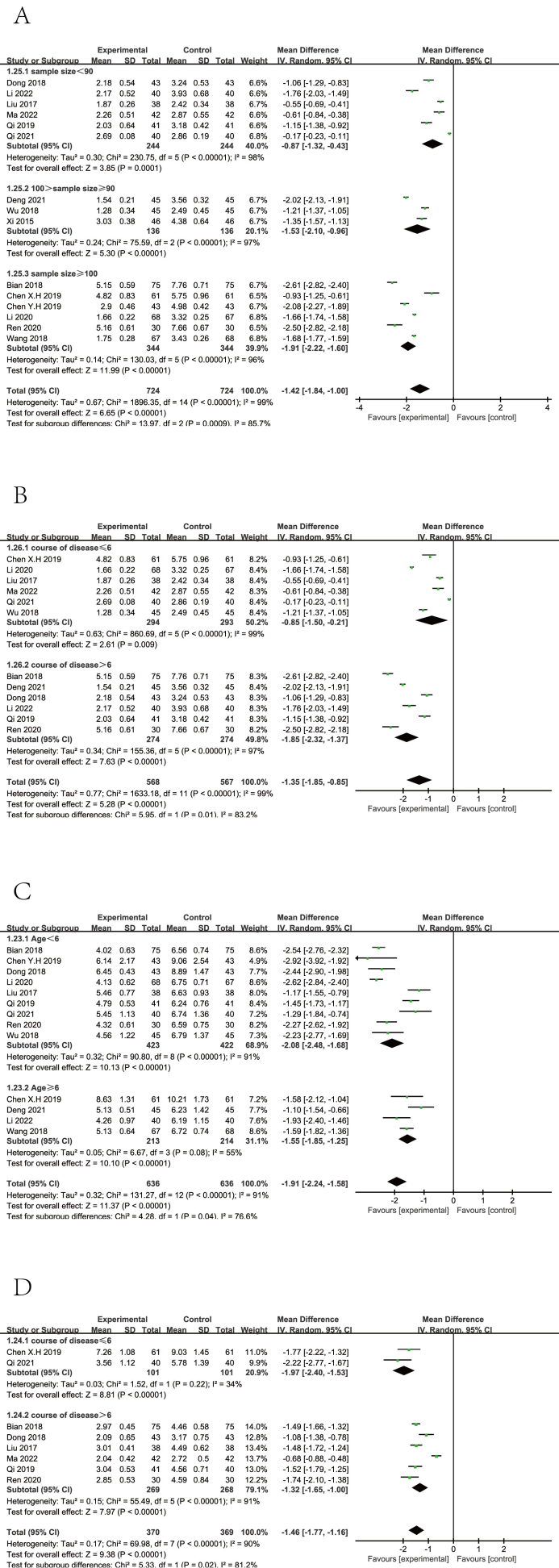


# **Supplementary Fig. 4 Regression plot of disappearance time of cough**

(A)Age;(B)Dosage;(C)Sample size;(D)Regional disparities between North and South China;(E)Course of disease;(F)The use of acupoint application.**
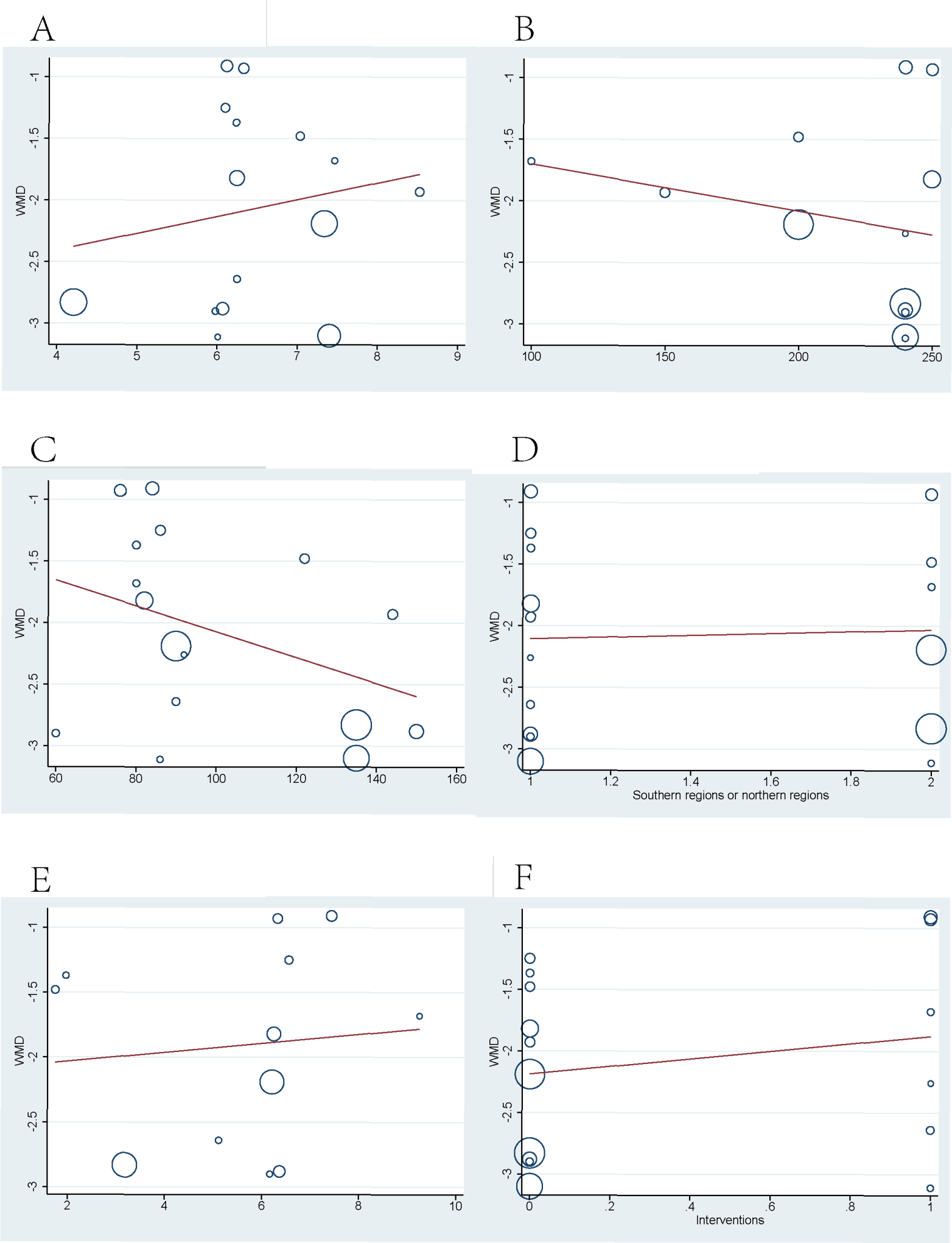
**

# **Supplementary Fig. 5 Regression plot of disappearance time of pulmonary rale**

(A)Age;(B)Dosage;(C)Sample size;(D)Regional disparities between North and South China;(E)Course of disease;(F)The use of acupoint application.


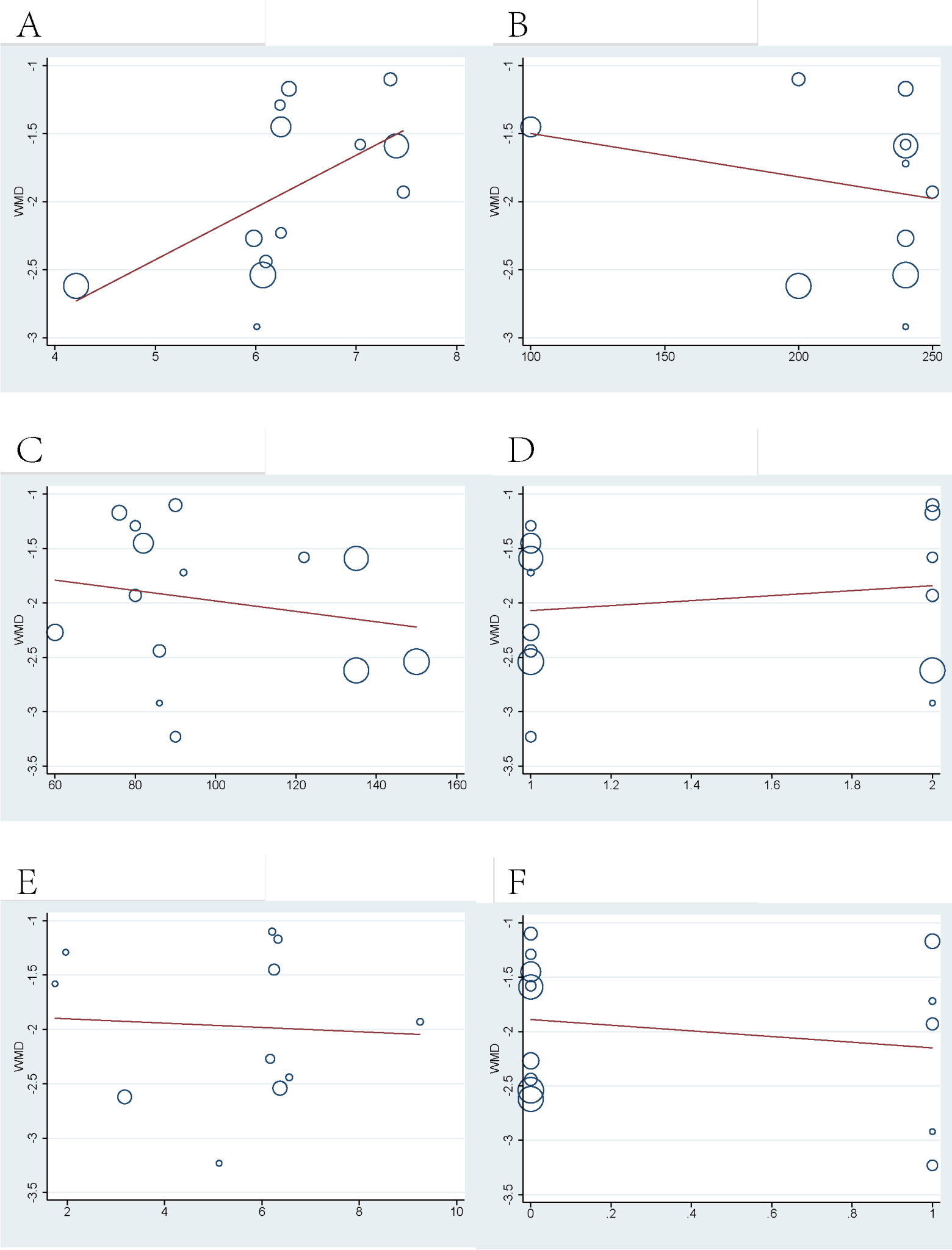


# **Supplementary Fig. 6** Regression plot of disappearance time of wheezing

(A)Age;(B)Dosage;(C)Sample size;(D)Regional disparities between North and South China;(E)Course of disease;(F)The use of acupoint application.


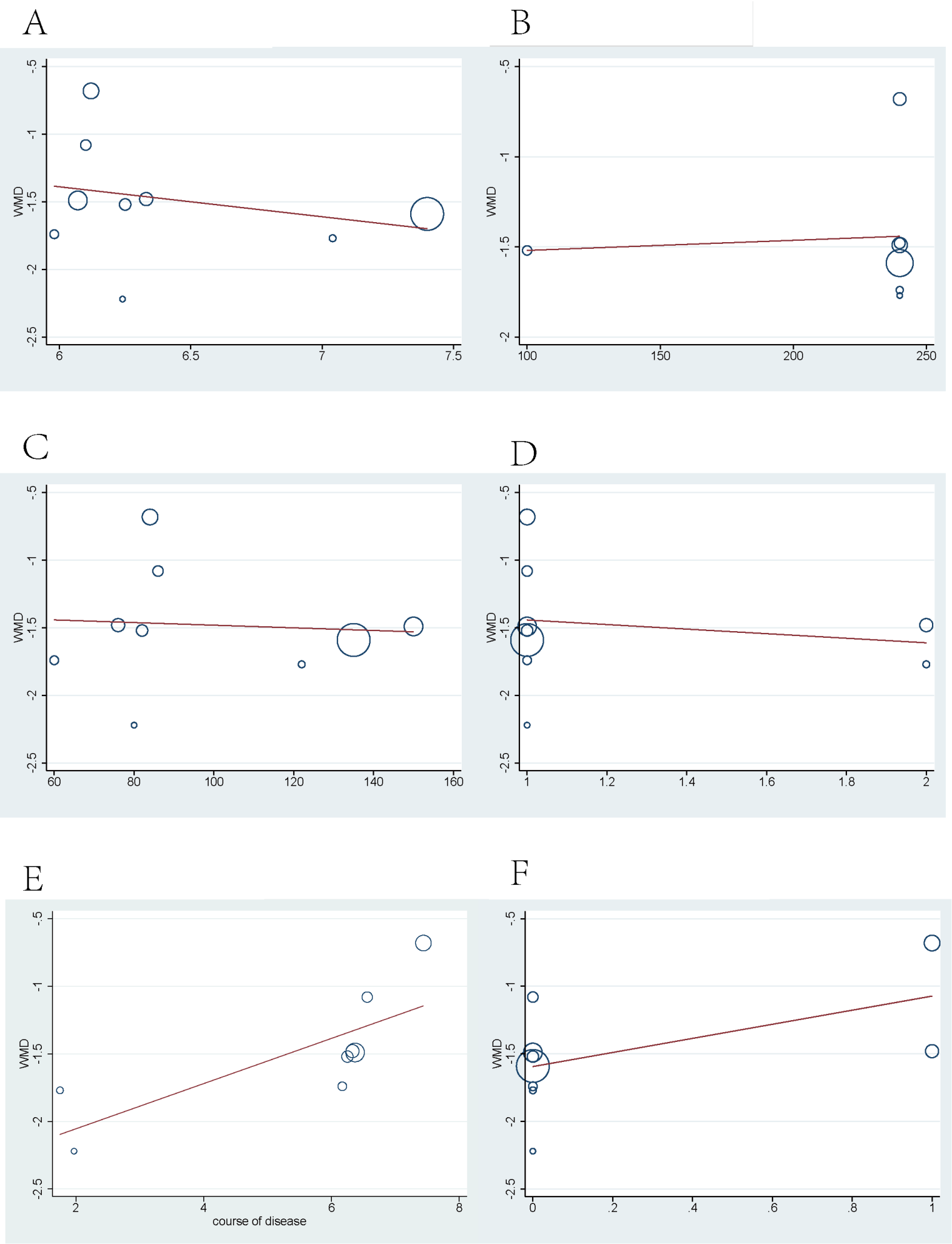


# **Supplementary Fig. 7 Forest plot of the length of hospital stay**


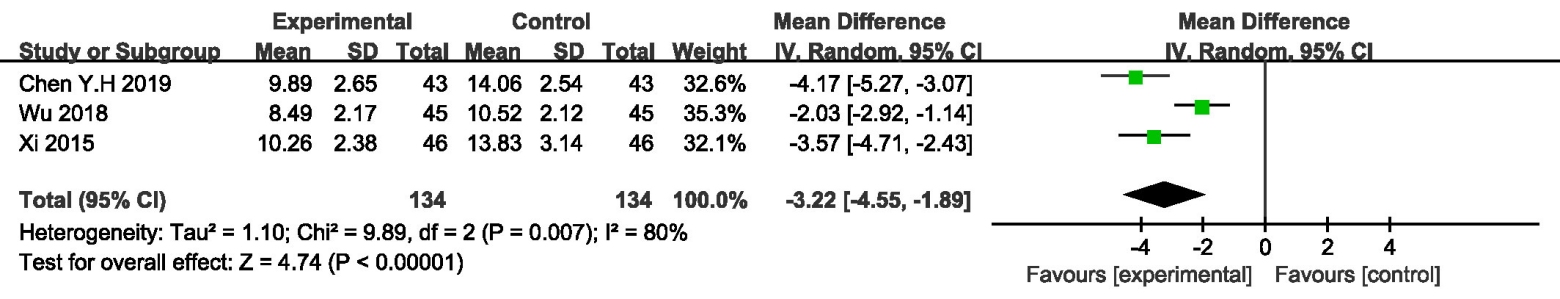


# **Supplementary Fig. 8 Forest plot of the T lymphocyte subsets**

(A)CD3+;(B)CD4+;(C)CD8+;(D)CD4+/CD8+.
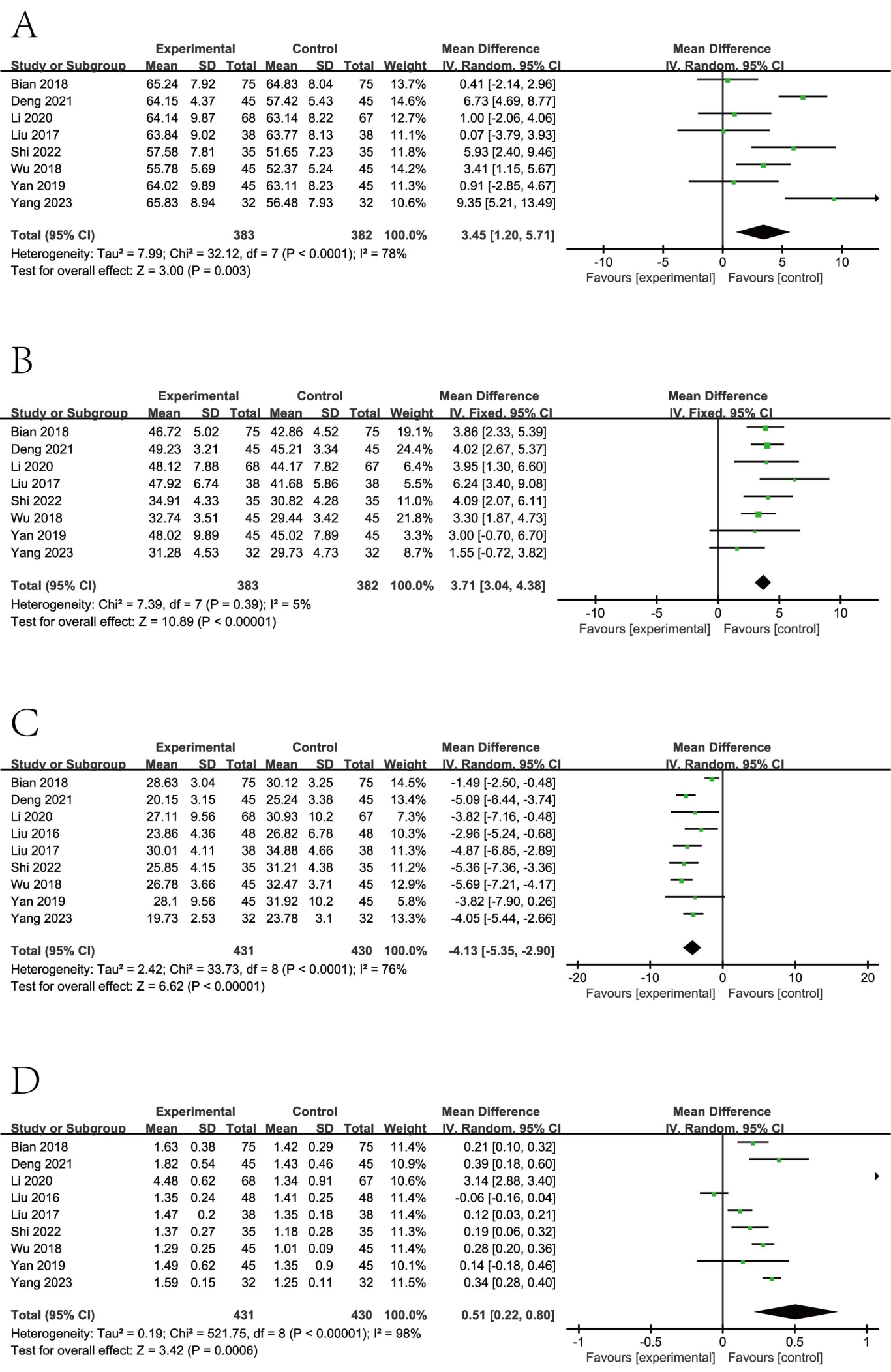


Supplementary Fig. 9 Forest plot of the inflammatory cytokines

(A)CRP;(B)IL-4;(C)IL-6;(D)IL-8;(E)IL-10;(F)TNF-α.


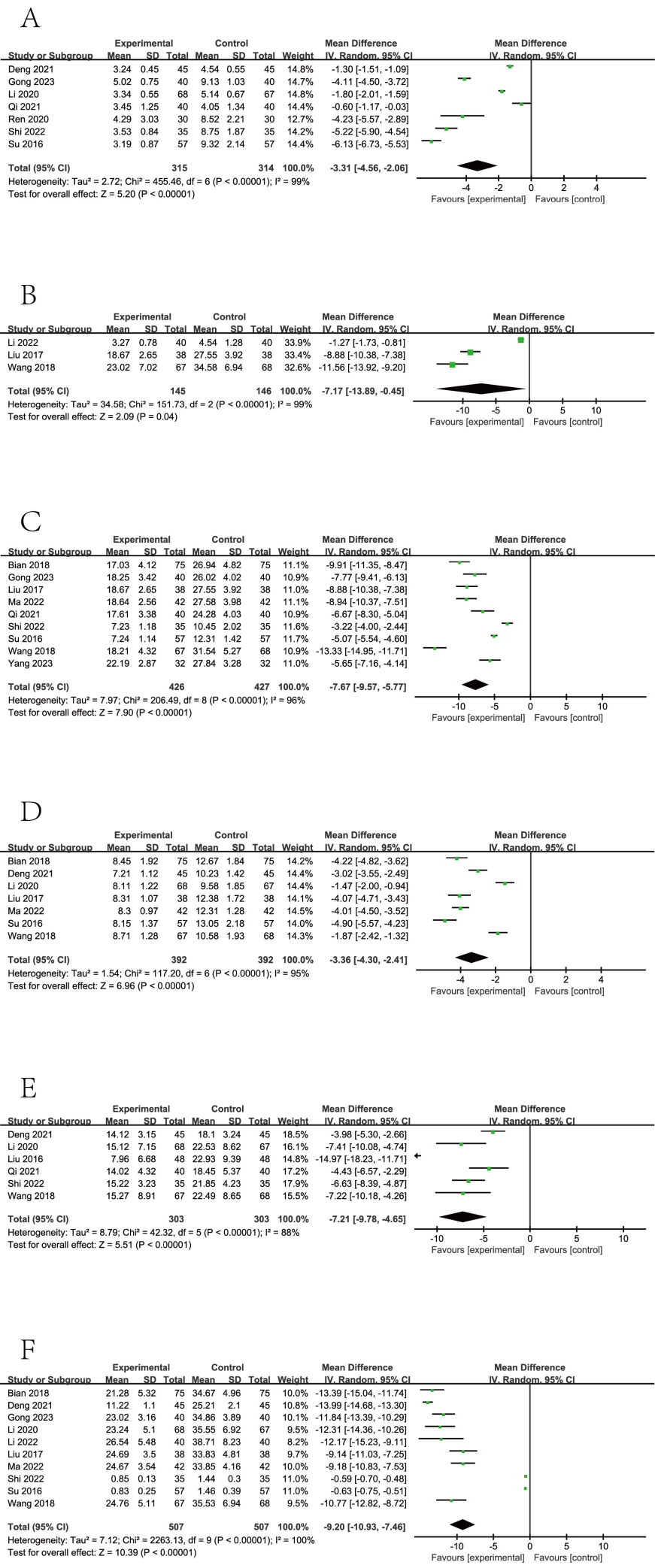


# **Supplementary Fig. 10 Forest plot of the adverse rate (safety)**

#
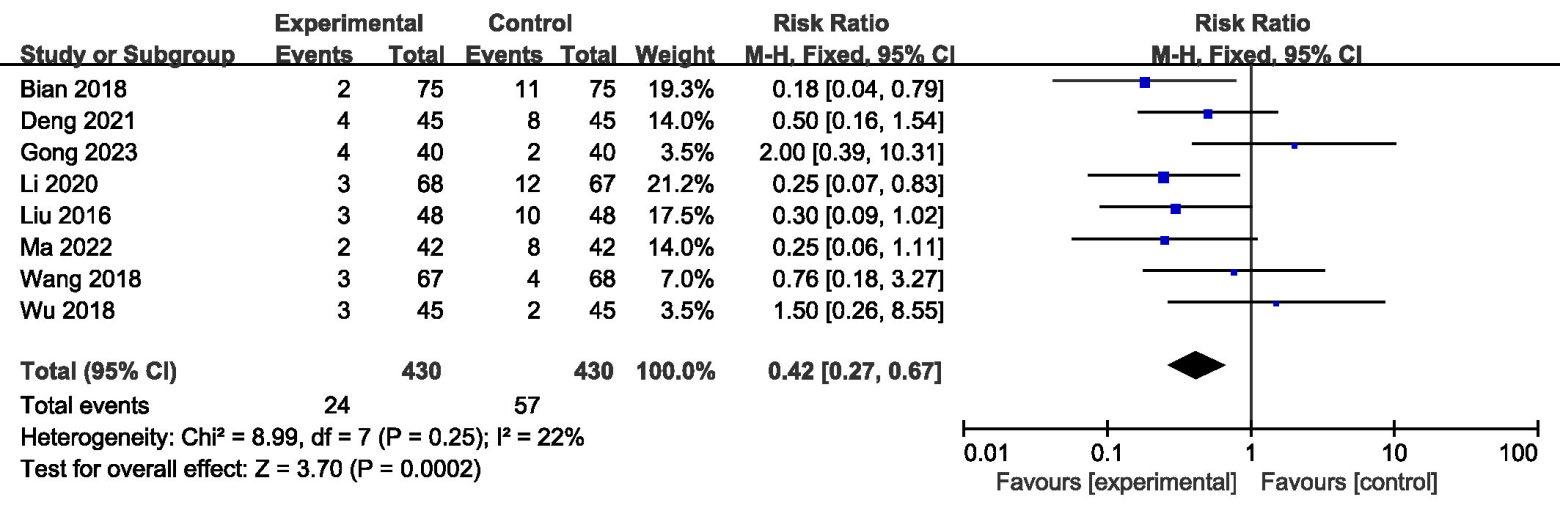

Supplement: Supplementary file 1 [file DataSheet1.docx]
